# The Hybrid Strategy of *Thermoactinospora rubra* YIM 77501^T^ for Utilizing Cellulose as a Carbon Source at Different Temperatures

**DOI:** 10.3389/fmicb.2017.00942

**Published:** 2017-05-29

**Authors:** Yi-Rui Yin, Zhao-Hui Meng, Qing-Wen Hu, Zhao Jiang, Wen-Dong Xian, Lin-Hua Li, Wei Hu, Feng Zhang, En-Min Zhou, Xiao-Yang Zhi, Wen-Jun Li

**Affiliations:** ^1^School of Life Sciences, Yunnan Institute of Microbiology, Yunnan UniversityKunming, China; ^2^Department of Cardiology, The First Affiliated Hospital of Kunming Medical UniversityKunming, China; ^3^State Key Laboratory of Biocontrol and Guangdong Provincial Key Laboratory of Plant Resources, School of Life Sciences, Sun Yat-Sen UniversityGuangzhou, China; ^4^Key Laboratory of Biopesticide and Chemical Biology, School of Life Sciences, Fujian Agriculture and Forestry UniversityFuzhou, China; ^5^Key Laboratory of Biogeography and Bioresource in Arid Land, Xinjiang Institute of Ecology and Geography, Chinese Academy of SciencesÜrümqi, China

**Keywords:** *Thermoactinospora rubra*, transcriptome, up-regulated cellulases, hybrid strategy, carbon source, different temperatures

## Abstract

*Thermoactinospora rubra* YIM 77501^T^ is an aerobic, Gram-positive, spore-forming and cellulose degrading thermophilic actinomycete isolated from a sandy soil sample of a volcano. Its growth temperature range is 28–60°C. The genomic sequence of this strain revealed that there are 27 cellulase genes belonging to six glycoside hydrolase families. To understand the strategy that this strain uses to utilize carbon sources such as cellulose at different temperatures, comparative transcriptomics analysis of *T. rubra* YIM 77501^T^ was performed by growing it with cellulose (CMC) and without cellulose (replaced with glucose) at 30, 40, and 50°C, respectively. Transcriptomic analyses showed four cellulase genes (*TrBG2, TrBG3, TrBG4*, and *ThrCel6B*) were up-regulated at 30, 40, and 50°C. The rate of gene expression of *TrBG2, TrBG3, TrBG4*, and *ThrCel6B* were 50°C > 30°C > 40°C. One cellulase gene (*TrBG1*) and two cellulase genes (*TrBG5* and *ThrCel6A*) were up-regulated only at 30 and 50°C, respectively. These up-regulated cellulase genes were cloned and expressed in *Escherichia coli*. The enzymatic properties of up-regulated cellulases showed a variety of responses to temperature. Special up-regulated cellulases *TrBG1* and *ThrCel6A* displayed temperature acclimation for each growth condition. These expression patterns revealed that a hybrid strategy was used by *T. rubra* to utilize carbon sources at different temperatures. This study provides genomic, transcriptomics, and experimental data useful for understanding how microorganisms respond to environmental changes and their application in enhancing cellulose hydrolysis for animal feed and bioenergy production.

## Introduction

Cellulose, as a main carbon source in the biosphere, is utilized by microorganisms and animals (Hungate, 1964; Batjes, 1996; Amundson, 2001). Completely digestion of cellulose involves multiple cellulases, like endo-1, 4-β-glucanases (EC 3.2.1.4), cellobiohydrolases or exo-1, 4-β-glucanases (EC 3.2.1.91), and β-glucosidases (EC 3.2.1.21; Lynd et al., 2002; Gilbert et al., 2008). For microorganisms, secretion of cellulases may be effected by substances, pH, ion, and temperature (Lynd et al., 2002; López-Contreras et al., 2004; Sohail et al., 2009; Deng and Zhang, 2015; Hakkinen et al., 2015; Chen et al., 2016). Temperature is one of the most important environmental factors (Lin et al., 2016), and it may impact utilization of cellulose as a carbon source from two aspects: (1) it could affect the activity and stability of cellulases, and (2) it might be a crucial factor for inducing some cellulases under special temperatures. The different cellulases of microorganisms have diverse activity and stability at the same or different temperatures (Pardo and Forchiassin, 1999), such as enzyme, EG5C from *Paenibacillus* sp. IHB B 3084 shows optimum activity below 45°C (Dhar et al., 2015), while enzymes, ThCel6A from *Thermobifida halotolerans* YIM 90462T and CelA from *Caldicellulosiruptor bescii* show maximum activity between 50 and 60°C and higher than 80°C, respectively (Brunecky et al., 2013; Yin et al., 2015). Some cellulases are stable only at a low temperature (Dhar et al., 2015), while some remain stable at a high temperature (Brunecky et al., 2013). Under high temperatures, *Thermobifida fusca* secretes thermo stable cellulolytic enzymes to degrade cellulose (Adav et al., 2011). However, the potential strategy of carbon source acquisition (such as cellulose) at different temperatures remains unclear for microorganisms.

With the passage of time, the environment is always changing, including the microenvironment that the microorganisms inhabit. Microorganisms have developed many strategies to successfully survive, and they occupy most of the habitats on Earth after long-term evolution (Jorge-Villar and Edwards, 2013), even in extreme niches. To adapt to different environmental conditions, microorganisms can adopt three possible strategies: (1) the active strategy (special enzymes were up-regulated under different conditions), (2) the passive strategy (the same enzymes were up-regulated under different conditions), or (3) a hybrid strategy (both the same kinds of and special kinds of enzymes were up-regulated under different conditions). Microorganisms could respond to the environmental factors (such as temperature) during the process of carbon source acquisition.

*Thermoactinospora rubra* YIM 77501^T^ is an aerobic, Gram-positive, spore-forming, and thermophilic actinomycete that was isolated from a sandy soil sample collected at the Tengchong National Volcanic Geological Park, Yunnan province, south-west China (Zhou et al., 2012). The growth temperature of strain YIM 77501^T^ ranges from 28 to 60°C and it grows optimally at 45–55°C (Zhou et al., 2012). The result of plate testing for cellulase production using a Congo red plate assay (Teather and Wood, 1982) demonstrated that *T. rubra* has the ability to degrade cellulose (Figure S1). The crude cellulase activity test from culture supernatants of *T. rubra* in 0.2% carboxymethyl cellulose (CMC) medium to stationary phase at different temperatures showed that the cellulase activity of the culture at 30 and 50°C were higher than culture at 40°C (Figure S2). Furthermore, comparing the effect of temperature on crude enzymes (culture supernatants of *T. rubra*) activity showed that the sample from 30°C had higher activity at 30–45°C, and the sample from 50°C had higher activity at 50–70°C. These clues clearly indicate that *T. rubra* could express cellulases with different enzymatic characteristics to digest cellulose as carbon source at different temperatures.

In this study, the draft genome of *T. rubra* YIM 77501^T^ was sequenced. The gene predication and function annotation revealed that there were 408 glycoside-hydrolase-encoding genes. The transcriptomes of *T. rubra* under different culture conditions (different temperatures: 30, 40, and 50°C; different carbon source: CMC sodium and glucose) were profiled as well. The cellulase genes, which were significantly up-regulated in the presence of CMC under different temperatures, were cloned and expressed heterogenetically. The enzymatic properties of these cellulases were characterized. This study shed light on the strategy of strain *T. rubra* for carbon source acquisition at different temperatures.

## Materials and methods

### Growth and genome sequencing

*T. rubra* YIM 77501^T^ (= DSM 45614^T^ = CCTCC AA 2011014^T^) was cultured on R_2_A medium (yeast extract, 0.5 g/l; peptone, 0.5 g/l; glucose 0.5 g/l; soluble starch, 0.5 g/l; casein acid hydrolysates, 0.5 g/l; sodium pyruvate, 0.5 g/l; K_2_HPO_4_, 0.3 g; MgSO_4_, 0.024 g/l) at 50°C. Genomic DNA was purified from 100 ml of mid exponential phase R_2_A cultures using a MasterPure Gram-Positive DNA Purification kit (Epicentre MGP04100) following the standard DNA isolation procedure recommended by the manufacturer with modifications (Wu et al., 2009). Genomic DNA was sequenced using Illumina technology (Bennett, 2004) at the Beijing Genomics Institute (BGI Tech Solutions, Shenzhen, China). After the raw sequences were trimmed and their quality filtered (*Q* > 30), the clean reads with high quality were assembled using de Bruijn graphs in SOAP *de novo* v.1.05 (http://www.seekbio.com/soft/2754.html) with the K-mer parameter set to 41 (Li et al., 2008) and draft genomes were generated. The SOAPaligner v.2.21 alignment tool (http://soap.genomics.org.cn/soapaligner.html#down2) was used to align these reads against the *de novo* scaffolds to map reads and account for single nucleotide errors (Gu et al., 2013). Glimmer v3.0 (Chen et al., 2011) was used for gene prediction in assembled sequences of strain *T. rubra*. The sequence data described here have been deposited in JGI IMG (Submission ID: 105093) and DDBJ/ENA/GenBank (Accession number: MSZZ00000000). Carbohydrate-active enzymes (CAZymes) of *T. rubra* were determined using the CAZymes Analysis Toolkit (http://mothra.ornl.gov/cgi-bin/cat/cat.cgi; Petit et al., 2015). The results of GHs (Glycoside hydrolase families) were analyzed using the HMMER software based on the Pfam database (http://pfam.xfam.org/; Finn et al., 2011).

### Transcriptome sample preparation and sequencing

*T. rubra* YIM 77501^T^ was cultured in a modified form of a previously described R_2_A medium containing the following (g/l): yeast extract, 0.5 g; peptone, 0.5 g; casein acid hydrolysates, 0.5 g; sodium pyruvate, 0.5 g; K_2_HPO_4_, 0.3 g; MgSO_4_, 0.024 g; with 2 g/l CMC sodium as the test sample (R_2_A-CMC medium) and with 2 g/l glucose as control (R_2_A-glucose medium), with the pH adjusted to 7.0 using KOH. The colony of *T. rubra* was inoculated to R_2_A-CMC and R_2_A-glucose media. The growth curves under different conditions were determined (Figure S3). After incubation to mid exponential phase (at 30°C 180 rpm for 7 days, 40°C 180 rpm for 3.5 days, and 50°C 180 rpm for 2 days), cells were harvested by centrifugation for 5 min at 12,000 × g at 4°C. Three independent biological replicates were performed for each condition. The cell biomass was washed with phosphate buffered saline, re-suspended in 100 μl TE buffer pH 8 (EMD Chemicals) containing 2 mg/ml lysozyme (Merck, USA), and incubated at 37°C for 40 min. Total RNA was isolated using the RNeasy RNA purification kit (QIAGEN, Germany) according to the manufacturer's instructions. Contaminating DNA was removed with RNase-free DNase I (QIAGEN, Germany). Ribosome RNA in total RNA preparation was removed by using the Ribo-Zero™ Magnetic Kit for Gram-Positive Bacteria (Epicentre, USA). The quality of RNA samples was assessed on the Agilent Bioanalyzer 2100 system. Library construction and Illumina sequencing (Illumina HiSeq™ 2500) were performed at the Beijing Novogene Biological Information Technology Co., Ltd., Beijing, China (http://www.novogene.cn/).

An RNA-seq analysis was performed according to the protocol recommended by the manufacturer (Illumina Inc.). Raw reads of fastq files were first processed through in-house Perl scripts. In this step, clean reads were utilized by removing reads containing adapter, reads containing poly-N and low quality reads from raw data. At the same time, the Q20, Q30, and GC contents of the clean data were calculated. All the following analyses were based on clean data with high quality. The reads from different conditions were mapped to the whole-genome of strain *T. rubra* using Bowtie 2.2.3 (Langmead and Salzberg, 2012). HTSeq v0.6.1 was used to count the read numbers mapped to each gene. Then, the FPKM of each gene was calculated based on the length of the gene and reads count mapped to this gene (FPKM, expected number of fragments per kilobase of transcript sequence per millions base pairs sequenced, considers the effect of sequencing depth and gene length for the reads count at the same time and is currently the most commonly used method for estimating gene expression levels; Trapnell et al., 2009). Here, FPKM > 1 means gene expression.

Differential expression analysis of two conditions (cultures in CMC media and glucose media) at the same temperature (three biological replicates per condition) were performed using the DESeq R package (1.18.0). DESeq provided statistical methods for determining differential expression in digital gene expression data using a model based on a negative binomial distribution. The resulting *p*-values were adjusted using the Benjamini and Hochberg approach for controlling false discovery rate. Genes with an adjusted *p* < 0.05 found by DESeq were assigned as differentially expressed (Wang et al., 2010).

### Gene cloning, expression, and purification of cellulases

The full-length sequences of cellulase genes were amplified from *T. rubra* genomic DNA. Chromosomal DNA of *T. rubra* was isolated using the Ezup Bacteria DNA Kit (Sangon Biotech, China) according to the manufacturer's instructions. Base on genome sequences, primers of cellulase genes (Table S1) were designed by using Primer Premier 5 (http://www.bioprocessonline.com/doc/primer-premier-5-design-program-0001). The complete ORFs of cellulase genes were amplified by PCR using the TransStar FastPfu Fly DNA Polymerase (TransGen Biotech, China). Amplification was performed for 34 cycles of 98°C for 20 s, 65°C for 20 s, and 72°C for 1 min, 72°C for 5 min with initial 2 min denaturation at 98°C. PCR products were ligated into pEASY-Blunt E1 vector (TransGen Biotech, China) and transformed into *Escherichia coli* DH5α. After sequencing verification, the entire cellulase gene was confirmed; positive recombinant vectors were transformed into *E. coli* BL21 (DE3) for cellulase gene expression.

Transformants were cultured overnight in LB culture medium with 100 μg/ml ampicillin at 37°C and 220 rpm. Then, 1 ml of the cells was added to 100 ml LB medium at 25°C and 220 rpm. During cultivation, isopropyl β-D-1-thiogalactoside (IPTG) was added to a final concentration of 1 mM at mid-exponential phase (OD600 ≈ 0.6) and followed by further incubation 8 h at 25°C with 220 rpm. Cells were harvested by centrifugation at 4,000 × g and suspended in 20 ml PBS buffer (pH 8.0).

After ultrasonic cell disintegration and centrifugation at 12,000 × g for 30 min at 4°C, cell-free extracts were applied to a Ni-chelating affinity column (GE, USA) because the proteins possess an N-terminal His-tag. Then, the extract was washed with five column volumes of buffer A (20 mM sodium phosphate, 0.3 M NaCl, pH 8.0), followed by 10 column volumes of buffer A with 20 mM imidazole, pH 8.0, and was eluted with buffer A with 200 mM imidazole, pH 8.0. The eluted protein was used for enzyme characterization.

### Enzyme assays and protein assays

The homogeneity of the purified enzyme was monitored by SDS-PAGE using 10% acrylamide gels. Proteins were visualized by Coomassie brilliant blue R-250 as described by Liu et al. (2010). Protein concentration was determined with a protein assay kit (Sangon Biotech, China) using bovine serum albumin as a standard. Activity against CMC was determined by measuring the release of reducing sugar, with 1% (w/v) CMC as substrate, by the 3,5-dinitrosalicylic acid (DNS) assay (Miller, 1959). One unit (U) of CMCase activity was defined as the amount of enzyme to release 1 μmol glucose-equivalent reducing sugars per minute. β-glucosidase activity was assayed using a 200 μl reaction mixture containing 2.5 mM p-nitrophenyl-β-D-glucopyranoside (pNPGlu) (Sigma, St. Louis, MO, USA). After 5 min of incubation at optimal temperature, the reaction was stopped by adding 0.6 ml of 1 M Na_2_CO_3_ (Yang et al., 2015). The p-nitrophenol was determined by monitoring the absorbance at 405 nm (Harnpicharnchai et al., 2009). One unit of β-glucosidase activity is equivalent to 1 μmol of p-nitrophenol released from the pNPGlu in 1 min. The effect of temperature was determined at different temperatures from 20 to 90°C in optimal pH. Thermal stability of enzymes was determined by incubating equivalents of purified enzyme solutions for varied length of time intervals at 30, 40, and 50°C. The residual activity was determined by the standard method.

## Results

### Genomic features of *T. rubra*

The draft genome sequence of *T. rubra* YIM 77501^T^ consisted of 191 scaffolds, with 8,233,369 bp. The GC content was 71.7% and the genome contained 8,114 coding sequences, with an average length of 914 bp. The general genomic features of *T. rubra* are listed in Table S2. Among the predicted genes, 60% (4867 genes) have been assigned a function, and 40% (3,247 genes) have an unknown function. In addition, genes encoding one rRNA operon were found in proximity to the origin of function, and there were 54 tRNAs (Table S2).

### Genes encoding carbohydrate-active enzymes

**Figure 1:**
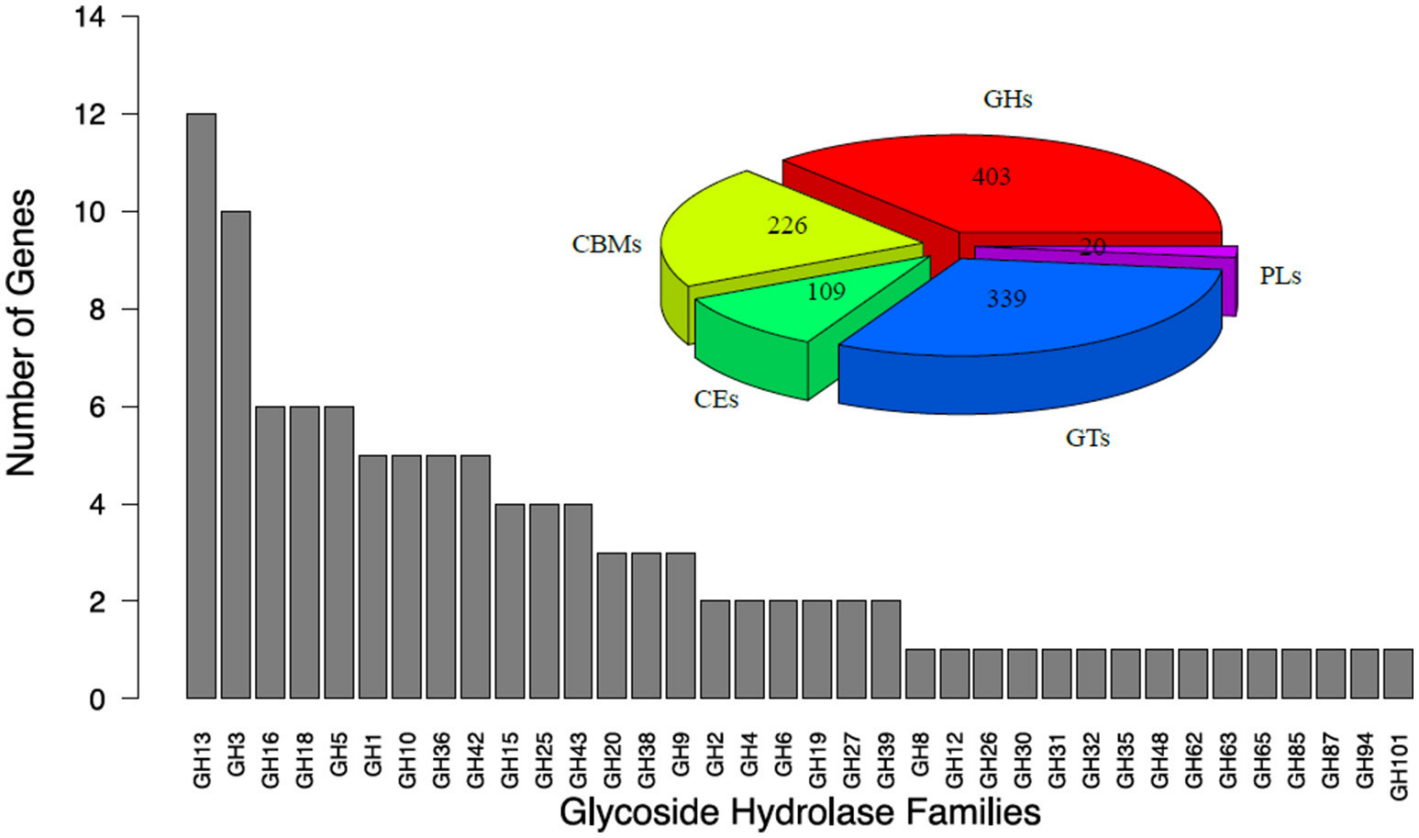
**Glycoside hydrolase (GHs) families of ***T. rubra*** YIM 77501^**T**^**. GHs, Glycoside Hydrolases; CBMs, Carbohydrate-Binding Modules; CEs, Carbohydrate Esterases; GTs, Glycosyl Transferases; PLs, Polysaccharide Lyases.

A CAZymes analysis was conducted to identify potential enzymes with plant cell-wall degradation ability. By applying this analysis, a total of 403 glycoside hydrolases (GHs) were distributed into 58 families, 226 carbohydrate-binding modules (CBMs) were distributed into 20 families, 20 polysaccharide lyases (PLs) were distributed into 7 families, 109 carbohydrate esterases (CEs) were distributed into 10 families, and 339 glycosyl transferases (GTs) were distributed into 26 families, are encoded in the genome of *T. rubra* (Figure 1). After analysis of GHs by using the HMMER software based on the Pfam database, 108 GH genes were distributed into 36 families (Figure 1). Twenty-seven cellulase genes were distributed to 6 GH families. The function of these 6 GH (GH1, GH3, GH5, GH6, GH9, and GH48) families may directly relate to cellulose digestion.

### Transcriptome sequencing and analyses

Global gene expression profiles of *T. rubra* cultured on R_2_A-glucose and R_2_A-CMC media under different temperatures (30, 40, and 50°C) were examined using transcriptome sequencing. Finally, there are 406.48 million clean reads and 50.81 GB of RNA-seq data in treated and control strains after quality filtering (Error rate = 0.01%, Q20 > 98%, Q30 > 95%). More than 99% of the clean reads were mapped to the *T. rubra* YIM 77501^T^ genome. The GC content of clean reads is within the scope of 68.2–69.2% for all samples (Table S3). All of the Pearson's correlations between biological replicates were >0.95 and indicated a high reliability of the experiment and rationality of sample selection (Figure S4). Comparing percentage of reads mapped to the genome regions at different temperatures, more reads were mapped to intergenic region at higher temperatures. Cultures of R_2_A-CMC media contained ~8.9, 12.3, and 21% reads mapping to intergenic regions at 30, 40, and 50°C, respectively. Cultures of R_2_A-glucose media contained ~10.8, 19.9, and 22.1% reads mapping to intergenic regions at 30, 40, and 50°C, respectively (Figure S5). These results revealed that intergenic regions might play some functions in adapting to higher temperatures for strain *T. rubra*.

**Figure 2:**
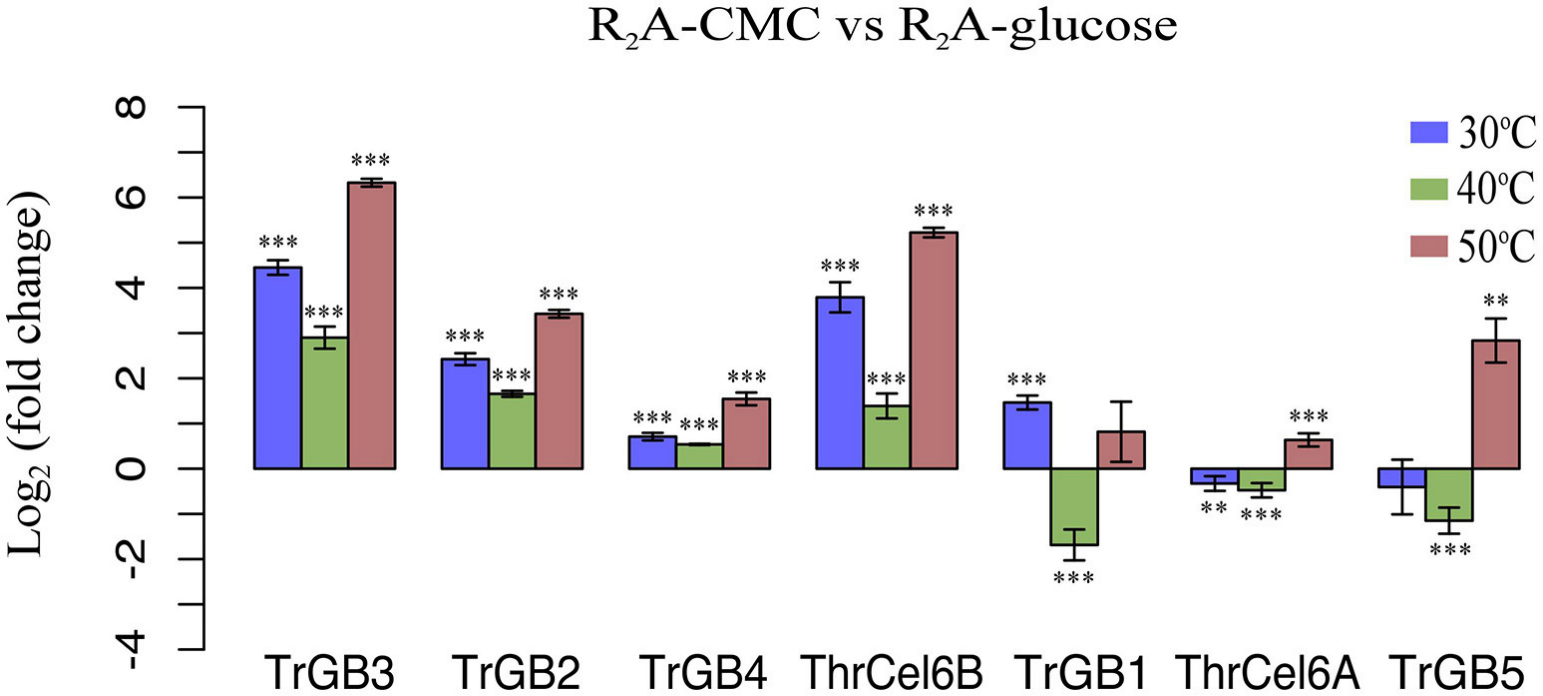
**Log2 of fold change in gene expression of ***T. rubra*** YIM 77501^**T**^ on R_**2**_A-CMC and R_**2**_A-glucose**. Blue bars show the expression of R_2_A-CMC cultures relative to R_2_A-glucose cultures at 30°C, green bars show the expression of R_2_A-CMC cultures relative to glucose (Glu) cultures at 40°C, red bars show the expression of R_2_A-CMC cultures relative to R_2_A-glucose cultures at 50°C. Values represent the mean of three biological replicates. Error bars show the standard deviation. ^***^p < 0.01; ^**^p < 0.05.

Total 18, 19, and 21 cellulase genes showed expression (FPKM > 1) at 30, 40, and 50°C, respectively. In these genes, seven up-regulated cellulase genes belonging to GH1, GH3, and GH6 were detected, comparing the cellulase genes expressions of CMC cultures with glucose cultures. This means that not all cellulase genes expressed during our culture conditions and the presence of CMC were no need for expression of some cellulase genes. Comparing the cellulase genes expressions of CMC cultures with glucose cultures at same temperature, four cellulase genes (*TrBG2, TrBG3, TrBG4*, and *ThrCel6B*) were up-regulated at 30, 40, and 50°C (*p* < 0.01). The rates of gene expression of *TrBG2, TrBG3, TrBG4*, and *ThrCel6B* were relatively higher at 50°C; on the contrary, they were relatively lower at 40°C. One cellulase gene (*TrBG1*) was especially up-regulated at 30°C (*p* < 0.01); two cellulase genes (*TrBG5* and *ThrCel6A*) were merely up-regulated at 50°C (*p* < 0.01; Figure 2). These findings suggested that culture temperature can affect cellulase gene expressions for *T. rubra*.

**Figure 3:**
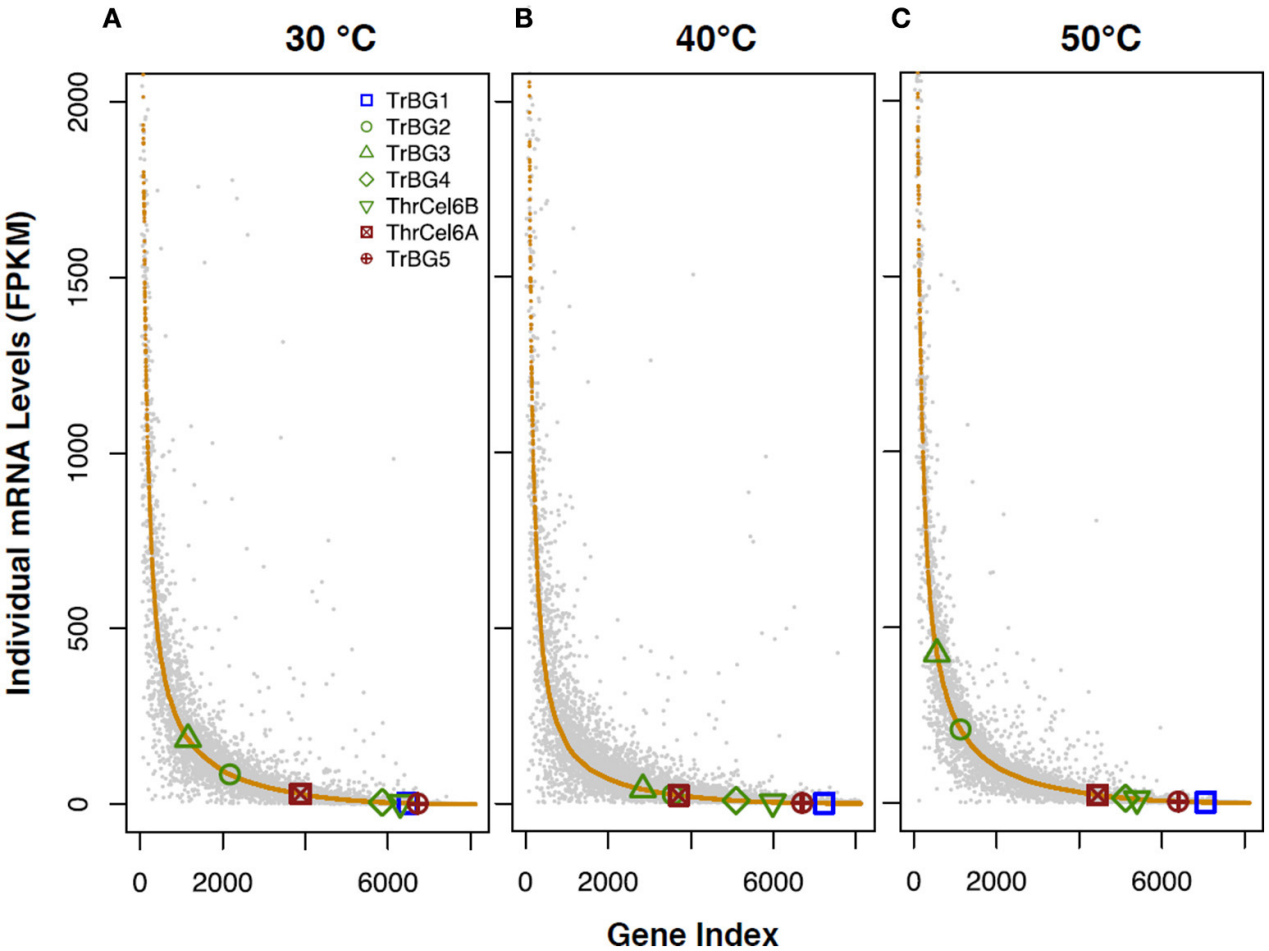
**Illustration of the variation in transcription levels of ***T. rubra*** YIM 77501^**T**^cultured in R_**2**_A-glucose (gray spots) and R_**2**_A-CMC (orange spots) media at different temperatures**. *T. rubra* YIM 77501^T^ was cultured at **(A)** 30°C, **(B)** 40°C, and **(C)** 50°C.

After data analysis, 7,084 and 7,132 genes expressed (FPKM > 1) when *T. rubra* was cultured at 30°C in R_2_A-CMC and R_2_A-glucose media, respectively; 7,009 and 6,839 genes were expressed (FPKM > 1) at 40°C in R_2_A-CMC and R_2_A-glucose media, respectively; 7,345 and 7,228 genes were expressed (FPKM > 1) at 50°C in R_2_A-CMC and R_2_A-glucose media, respectively, (Figure 3, Table S4). The expression of different genes in *T. rubra* showed high fluctuation when cultured at different temperatures on R2A-CMC and R2A-glucose media. High expression genes was observed (FPKM > 60), when *T. rubra* was cultured at 30 and 50°C as compared at 40°C. The genes 2,725 and 2,750, 2,231 and 2,543, 2,835 and 2,852 showed high expression (FPKM > 60) when *T. rubra* was cultured at 30, 40, and 50°C on R2A-CMC and R2A-glucose media, respectively (Table S4).

The genes expression profiles displayed remarkable differences when *T. rubra* was cultured at different temperatures in the same media (R_2_A-CMC or R_2_A-glucose). Comparing 30°C with 40°C, 4,473 genes (2,253 genes up-regulated and 2,220 genes down-regulated) were significant differentially expressed in R_2_A-CMC media and 3,894 genes (2,093 genes up-regulated and 1,801 genes down-regulated) were significant differentially expressed in R_2_A-glucose media. Comparing 30°C with 50°C, 4,918 genes (2,489 genes up-regulated and 2,429 genes down-regulated) were significant differentially expressed in R_2_A-CMC medium and 4,988 genes (2,510 genes up-regulated and 2,478 genes down-regulated) were significant differentially expressed in R_2_A-glucose medium. Comparing 40°C with 50°C, 4,293 genes (2,219 genes up-regulated and 2,074 genes down-regulated) were significant differentially expressed in R_2_A-CMC medium and 3,760 genes (1,805 genes up-regulated and 1,955 genes down-regulated) were significant differentially expressed in R_2_A-glucose medium (Figure S6A). The functions of these differentially expressed genes at different temperatures are mainly reflected in the biological process, cellular component, and molecular function (Figure S6B).

**Figure 4:**
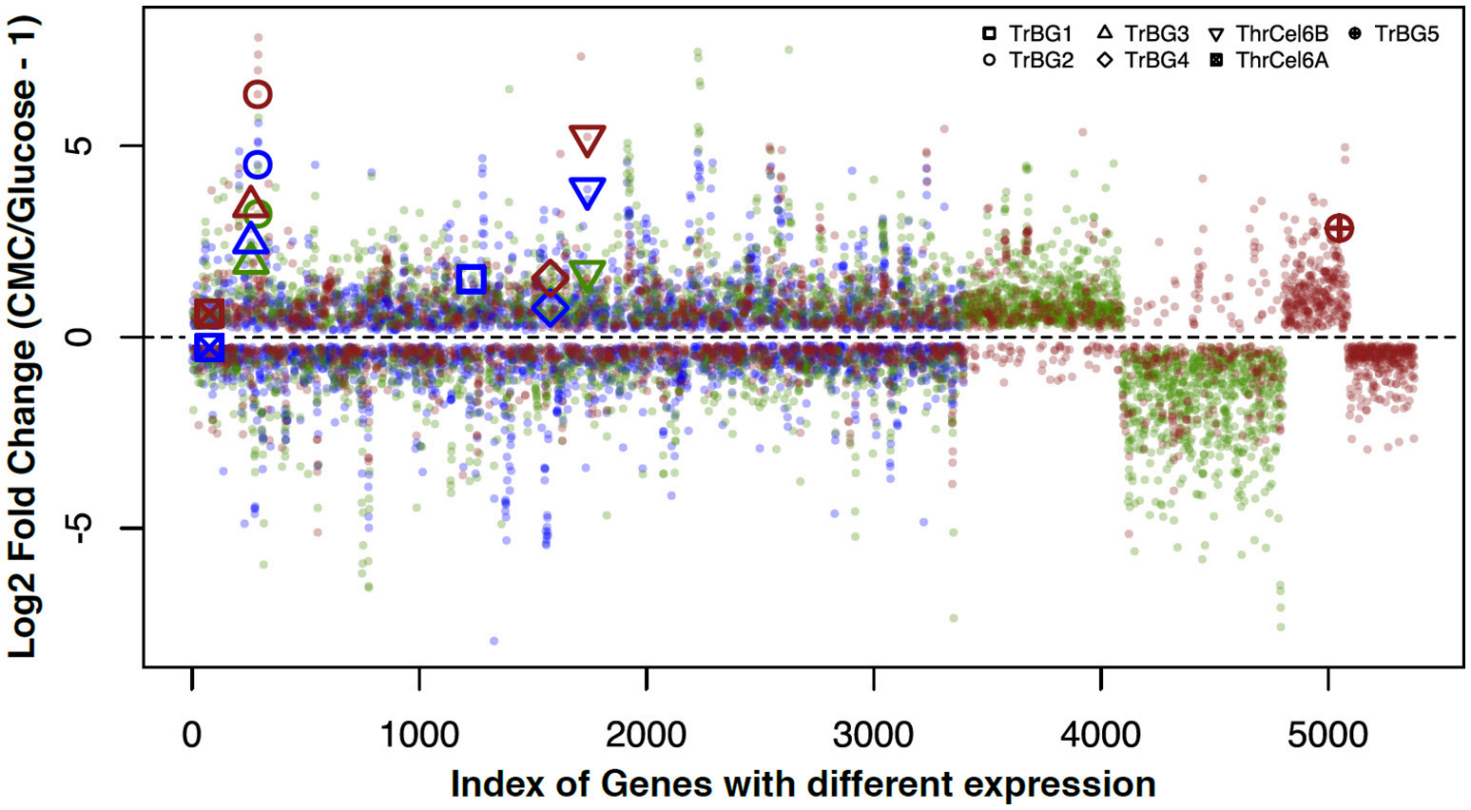
**Correlation between the log_**2**_ fold changes of genes that were differentially expressed in response to CMC and Glucose at different temperature (30, 40, and 50°C)**. Genes that showed log_2_ fold change (CMC/Glucose-1) of > 0 or < 0 in the same culture temperature were included for comparison. Blue spots show cultures at 30°C; green spots show cultures at 40°C; red spots show cultures at 50°C.

Comparing genes expression profiles of samples from R_2_A-CMC and R_2_A-glucose media at different temperatures, 3,398 genes (1,706 up-regulated genes and 1,692 down-regulated genes) were found at 30°C, 3,560 genes (1,929 up-regulated genes and down-regulated 1,631 genes) were found at 40°C, and 2,869 genes (1,412 up-regulated genes and 1,457 down-regulated genes) were found at 50°C. Among these differentially expressed genes at different temperatures, 1,278 genes were up- and down- regulated at all temperatures, and 732, 504, and 577 genes were specifically expressed at 30, 40, and 50°C, respectively (Figure 4, Figure S7). This also showed more genes were specific expression when *T. rubra* is cultured at 30 and 50°C than at 40°C.

### Cloning, expression, and enzyme activity of up-regulated cellulases

**Figure 5:**
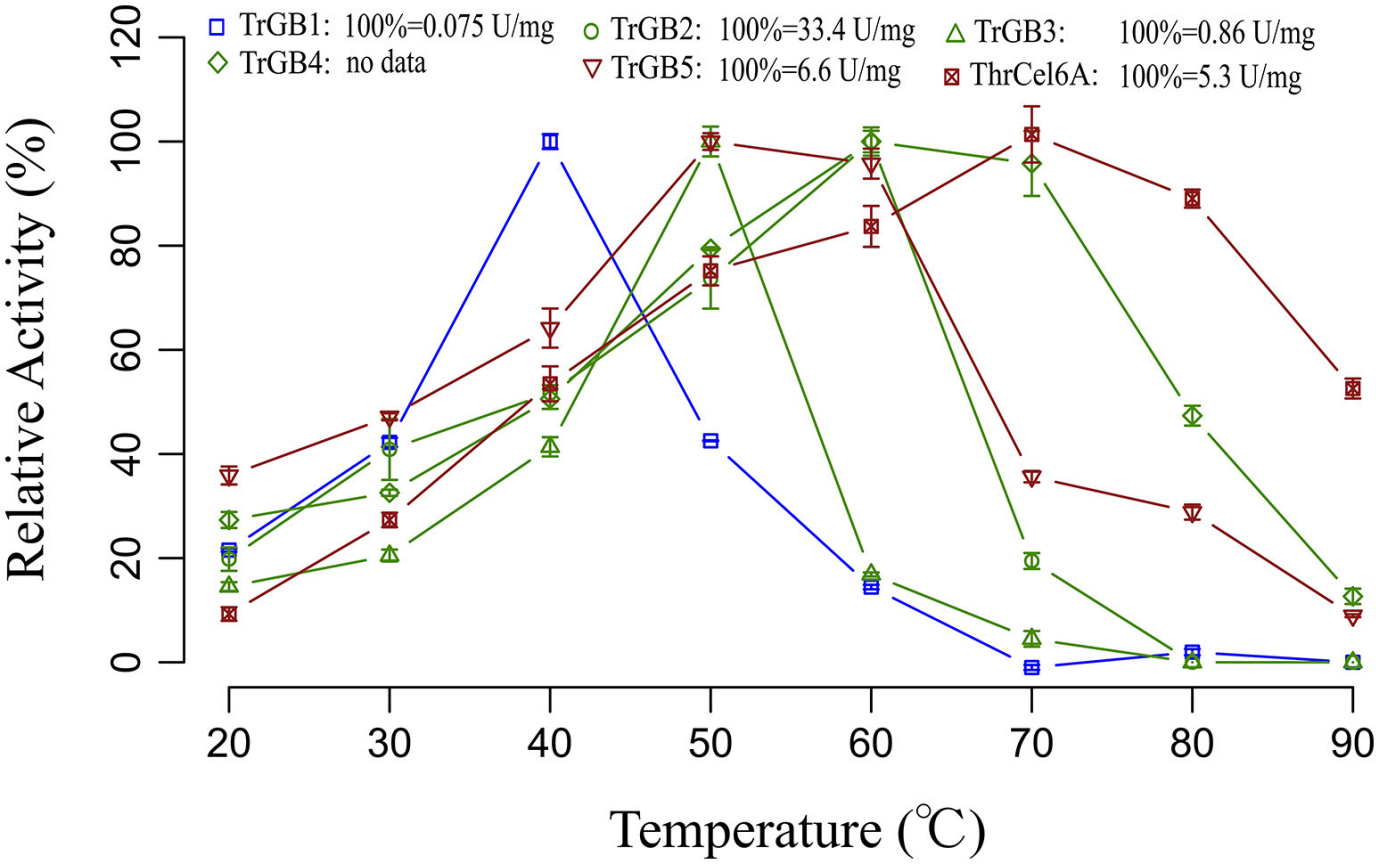
**Thermo activity profiles for cellulases**. Data points are initial rates of activity at a given temperature expressed as a proportion of the highest rate. The cellulase genes were up-regulated only at 30°C (blue); the cellulase genes were up-regulated at 30, 40 and 50°C (green); the cellulase genes were up-regulated only at 50°C (red). For TrBG1, 100% = 0.075 U/mg; TrBG2, 100% = 33.4 U/mg; TrBG3, 100% = 0.86 U/mg; TrBG4, no data; TrBG5, 100% = 6.6 U/mg; ThrCel6A, 100% = 5.3 U/mg. The error bars show the standard deviations from three measurements.

*TrBG2, TrBG3, TrBG4, ThrCel6B, TrBG1, TrBG5*, and *ThrCel6A* were cloned and expressed in *E. coli* BL21. TrBG1, TrBG2, TrBG3, TrBG1, TrBG5, and ThrCel6A were purified (Figure S8) and their enzyme activities were tested (Figure S9). The kinetic parameters of these enzymes are shown in Table S5. The enzyme activities of TrBG4 was tested using crude enzymes. Based on experimental results, TrBG1, TrBG2, TrBG3, TrBG4, and TrBG5 showed β-glucosidase enzyme activity; ThrCel6A showed CMCase enzyme activity. The optimal temperatures of TrBG1, TrBG2, TrBG3, TrBG4, TrBG5, and ThrCel6A were 40, 60, 50, 60–70, 50–60, and 70°C, respectively. TrBG1 showed high relative enzyme activity at 30°C (~40%). TrBG2, TrBG3, and TrBG4 showed more than 20% relative enzyme activity at 30–50°C ThrCel6B cannot be expressed in *E. coli* (by expression vector). TrBG5 and ThrCel6A showed high relative enzyme activity at 50°C (~100 and 78%, respectively; Figure 5).

**Figure 6:**
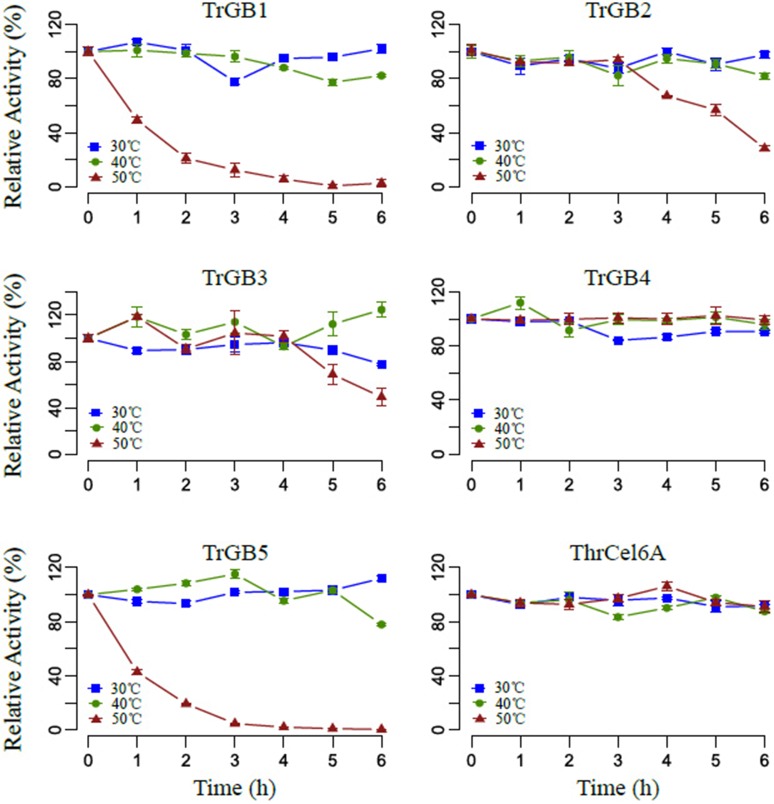
**The effect of temperature on stability**. Cellulases were incubated at temperatures 30°C (blue), 40°C (green), 50°C (red) for 0–6 h, and the remaining activities were assayed at optimal conditions. For TrBG1, 100% = 0.075 U/mg; TrBG2, 100% = 33.4 U/mg; TrBG3, 100% = 0.86 U/mg; TrBG4, no data; TrBG5, 100% = 6.6 U/mg; ThrCel6A, 100% = 5.3 U/mg. The error bars show the standard deviations from three measurements.

Comparing effects of temperatures on enzyme stability, TrBG1, TrBG2, TrBG3, TrBG4, TrBG5, and ThrCel6A kept more than 70% relative enzyme activity after incubating at 30 and 40°C for 6 h. TrBG2 and TrBG3 lost more than 50% relative enzyme activity after incubating at 50°C for 6 h, while TrBG1 and TrBG5 lost most enzyme activity (more than 80%) after incubating at 50°C for 3 h. These results revealed TrBG1 and TrBG5 may be unstable at 50°C *in vitro*. TrBG4 and ThrCel6A were stable (kept more than 90% relative enzyme activity) after incubating at 50°C for 6 h (Figure 6).

## Discussion

Research has shown that some potential functions of microorganisms, such as degradation ability of carbon sources, implemented by special functional genes can be predicted based on genomic data analysis (Denger et al., 2006; Klippel et al., 2011; Wibberg et al., 2016). Numbers of genes encoding carbohydrate-active enzymes include more than 100 GHs were found in the genome of *T. rubra*, suggesting that this strain can convert cellulose as its own carbon source into biomass. Our results demonstrated that strain *T. rubra* has many choices of enzymes available to degrade cellulose at different temperatures.

Transcriptome analysis showed that temperature can affect the transcription of *T. rubra*. The clean reads of transcriptome sequencing displayed more reads were mapped to intergenic regions from the lower temperature to the higher temperature (30–50°C). The intergenic region may play a regulatory function in gene expression for some microorganisms (Beneke et al., 1995), e.g., *lac* operator. It demonstrated that some intergenic region sequences were increasingly transcribed with the rise of temperature, and they might regulate different gene expression to respond to temperatures. After transcriptome analysis, more genes showed high expression (FPKM > 60) when *T. rubra* was cultured both at 30 and 50°Ccompared with at 40°C. This could be attributed to 30 and 50°C being nearer the growth temperature range (28–60°C) of *T. rubra* (Zhou et al., 2012). Under the stress of lower or higher temperatures, microorganisms will overexpress some special proteins, such as cold shock proteins (CSPs) or heat shock proteins (HSPs), to protect themselves (Kondo and Inouye, 1994; Yin et al., 2012). In low temperature, secondary structures in mRNA would obstruct coupling processes of transcription and translation (El-Sharoud and Graumann, 2007). In *E. coli*, CSPs act as transcription anti-terminators or translational enhancers to destabilize RNA secondary structure (Nakaminami et al., 2006). Compared with culturing at 40 and 50°C, CSPs were up-regulated when *T. rubra* was cultured at 30°C (Figure S10A). This suggested that CSPs of *T. rubra* may function as an RNA chaperone to destabilize secondary structures and may be involved in regulating translation under lower temperatures. Heat shock proteins (HSPs) exist in all organisms and are important for stress tolerance (such as high temperature, oxidative, acid, and alkali stress) for microorganisms (Krajewski et al., 2014). When *T. rubra* was cultured at 50°C, HSP (*dnaK*) was up-regulated compared with culturing at 30 and 40°C (Figure S10B).

Comparing genes expression profiles when *T. rubra* was cultured at different temperatures, more genes were up-regulated at lower temperatures than higher temperatures (30°C > 40°C, 30°C > 50°C). These results indicated that *T. rubra* may need to express more genes to live at a lower temperature. Global transcriptome analysis of *Lactococcus garvieae* strains also found more up-regulated genes at 18°C than at 37°C (Aguado-Urda et al., 2013) and demonstrated that different temperatures induced various gene expression changes in *T. rubra*.

Not all cellulase genes of *T. rubra* expressed and up regulated in presence of CMC. It revealed that expression of some cellulase genes may need some special induced conditions and there may be expression of some cellulase genes under presence of other carbon source, like glucose. For *Stachybotrys microspore*A19, carbon sources and the pH of the culture medium can direct a differential induction of various cellulases (such as endoglucanases and beta-glucosidases; Ben Hmad et al., 2014).

Temperature is an important environmental factor, and it determined the performance and stability of the enzyme used when utilizing a carbon source (Lin et al., 2016). For some microorganisms, temperature can affect the secretion of cellulases in the presence of cellulose substances, and this results in different cellulase enzyme activities at different temperatures (Sohail et al., 2009). *T. fusca*, as a thermophilic actinomycete, secretes thermo stable cellulolytic enzymes to digest cellulose at a high temperature (50°C) (Adav et al., 2011). Here, some cellulase genes were up-regulated by *T. rubra* when presented with CMC at all culture temperatures. In particular, other cellulase genes were up-regulated in just one temperature when presented with CMC. Based on the above-mentioned hypotheses, *T. rubra* used a hybrid strategy (combining the active strategy with the passive strategy) to utilize carbon sources at different temperatures. Comparing CMC cultures with glucose cultures, the expression of cellulase genes showed different patterns and levels at different temperatures. Four cellulase genes (*TrBG2, TrBG3, TrBG4*, and *ThrCel6B*) appeared to be up-regulated at three temperature conditions significantly, and one (*TrBG1*) and two (*TrBG5* and *ThrCel6A*) cellulase genes were significantly up-regulated only at 30 and 50°C, respectively. Moreover, the expression levels of up-regulated cellulase genes (such as *TrBG2, TrBG3, TrBG4*, and *ThrCel6B*) at 30 and 50°Cwere higher than at 40°C. These results correspond with cellulase activity of culture supernatants (Figure S2) and reveal that *T. rubra* can regulate its cellulase gene expression during the process of utilizing carbon source at different temperatures.

The enzymatic properties analysis of these up-regulated cellulase genes that were cloned from *T. rubra* showed a variety of responses to temperature. *TrBG2, TrBG3*, and *TrBG4* were up-regulated cellulase genes at 30, 40, and 50°C and were stable with more than 40% relative enzyme activity at 40°C. However, TrBG2 and TrBG3 had low activity (<30%) at 30°C and were not stable at 50°C after 6 h. Therefore, in light of evolution, extra cellulases may appear at lower temperatures (30°C) or higher temperatures (50°C). *TrBG1*is a special up-regulated cellulase gene at 30°C, and it has high (>50%) relative enzyme activity at 30°C. *ThrCel6A* is also a special up-regulated cellulase gene at 50°C and was stable at 50°C after 6 h. TrBG5 is a special up-regulated cellulase gene at 50°C, and its optimal temperature was 50°C. However, no signal peptide was predicted by using SignalP 4.1 Server (http://www.cbs.dtu.dk/services/SignalP/, Table S5) for TrBG5, and it is not as stable as ThrCel6A at 50°C. It almost lost enzyme activity after incubating at 50°C for 3 h *in vitro* (Figure 6). DnaK, a main HSP in bacteria (Flaherty et al., 1991), was up-regulated in *T. rubra* at 50°C (Figure S10B), and it may protect proteins including TrBG5 at high temperatures *in vivo*. The results of these regulations ensured an adequate supply of carbon source for the growth and reproduction of *T. rubra*. Transcriptome data and up-regulated cellulases properties were consistent with the cellulase activity of culture supernatants of strain *T. rubra* (Figure S2). Compared with the culture at 40°C, there were higher levels and extra up-regulated cellulase genes at 30 and 50°C. These extra up-regulated cellulase genes showed functional adaptation for 30 or 50°C.

Both enzymatic properties and gene expression levels of cellulases displayed adaption to temperature. The reason that higher levels and more numbers of cellulase genes were up-regulated at lower or higher temperatures may be explained by the effect of temperature to enzyme. As we know, temperature can affect the activity and stability of an enzyme (Pardo and Forchiassin, 1999). Most enzymes are stable in lower temperatures but have low enzyme activity. However, at higher temperature, they have higher enzyme activity and are not stable. When the enzymes perform biological functions, microorganisms need to balance the stability and activity of enzyme. The results of these regulations show that increased enzyme gene expression and up-regulated special enzyme genes at lower or higher temperatures may maintain biological functions of enzymes economically and efficiently for the microorganism at different temperatures. In addition to temperature, microorganisms can also change the expression of cellulase genes on other environmental factors, such as substrate, pH, and ions to utilize carbon source (Lynd et al., 2002; López-Contreras et al., 2004; Deng and Zhang, 2015; Hakkinen et al., 2015; Chen et al., 2016). The expression level of different cellulase genes can be changed by various substrates (López-Contreras et al., 2004). Ambient pH can regulate expression of cellulase and hemicellulase genes by the transcriptional regulator (PACI) for *Trichoderma reesei* (Hakkinen et al., 2015). After a long period of evolution and development, microorganisms have evolved diverse strategies to overcome the changing environment.

Over billions of years of evolution, microorganisms have created special strategies for environmental change. When a microorganism uses cellulose as a carbon source, cellulose induces several metabolic pathways (Adav et al., 2011). While, further research is needed for membrane transport and signal transduction, *T. rubra* YIM 77501^T^ processes environmental information via different strategies to utilize carbon source at different temperatures.

## Conclusions

To understand the strategies of carbon source acquisition, such as cellulose, in the environment at different temperatures for microorganisms, comparative transcriptomics of *T. rubra* grown with cellulose (CMC) and without cellulose (replace with glucose) at 30, 40, and 50°Cwere studied. The differences of the transcriptome analysis approach revealed that a hybrid strategy was used by *T. rubra* to utilize carbon sources at different temperatures. Higher levels and more numbers of cellulase genes were up-regulated at lower (30°C) or higher (50°C) temperatures than at a middle temperature (40°C). The enzymatic properties of up-regulated cellulase genes showed a variety of responses to temperature. Special up-regulated cellulases TrBG1 and ThrCel6A displayed temperature acclimation. These results may mean *T. rubra* has evolved an economical and efficient strategy to utilize carbon sources at different temperatures. This study provides genomic, transcriptomics, and experimental data useful for understanding how microorganisms respond to environmental changes and for their application in enhancing cellulose hydrolysis for animal feed and bioenergy production.

## Author contributions

WL, YY, and XZ designed research and project outline. YY, ZJ, and WX performed growth and genome sequencing. XZ, EZ, and FZ performed transcriptome sample preparation and sequencing. QH, LL, and WH provided the gene cloning, expression and purification of cellulases. ZM, FZ, and YY performed enzyme assays and protein assays. WL, YY, and XZ drafted the manuscript. All authors read and approved the final manuscript.

### Conflict of interest statement

The authors declare that the research was conducted in the absence of any commercial or financial relationships that could be construed as a potential conflict of interest.
