# Supplementary material for: The Hybrid Strategy of Thermoactinospora rubra YIM 77501T for Utilizing Cellulose as a Carbon Source at Different Temperatures
Source: Front Microbiol. 2017 May 29;8:942. doi: 10.3389/fmicb.2017.00942 (PMC5447088; doi:10.3389/fmicb.2017.00942)

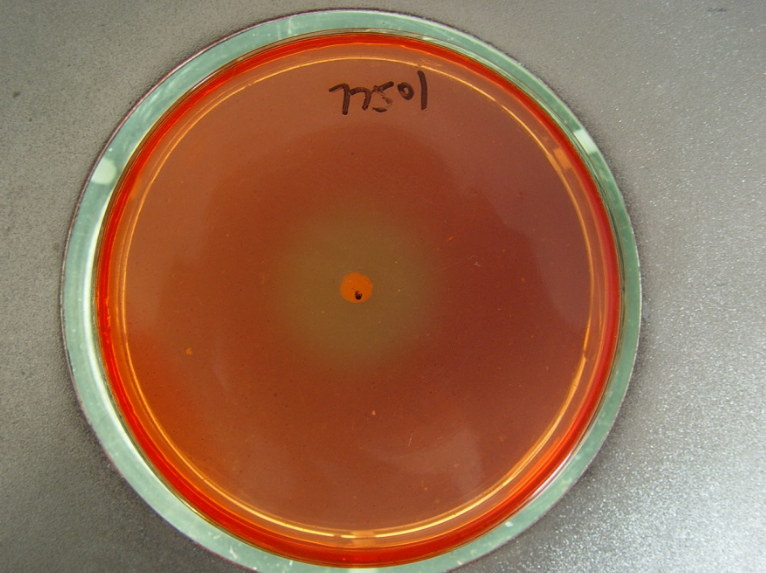

Supplement: Supplementary file 1 [file Presentation1.PDF]
