# Supplementary figures and images for: The Hybrid Strategy of Thermoactinospora rubra YIM 77501T for Utilizing Cellulose as a Carbon Source at Different Temperatures
Source: Front Microbiol. 2017 May 29;8:942. doi: 10.3389/fmicb.2017.00942 (PMC5447088; doi:10.3389/fmicb.2017.00942)

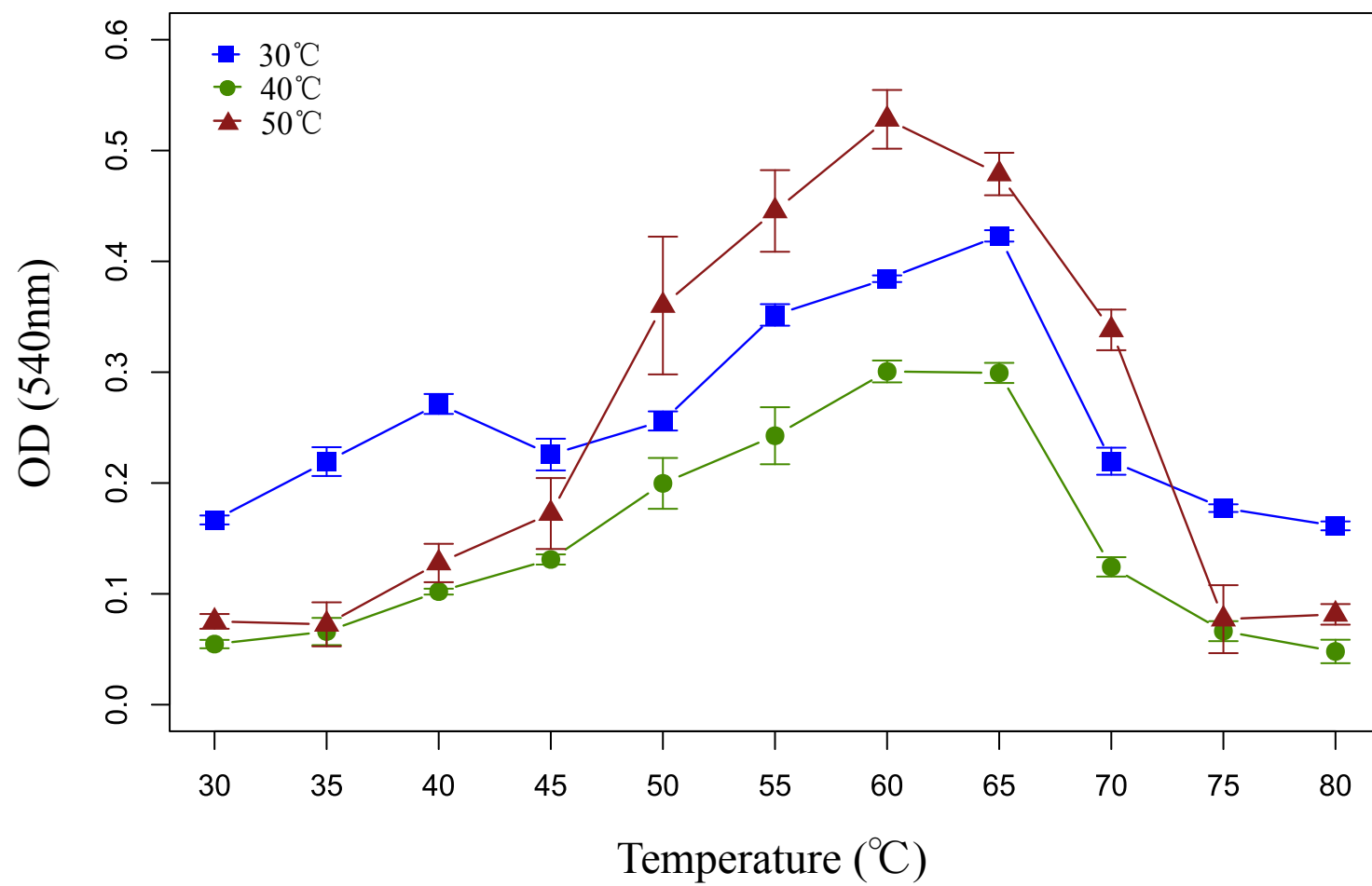

Supplement: Supplementary file 2 [file Presentation2.PDF]

Growth on R<sub>2</sub>A-glucose media

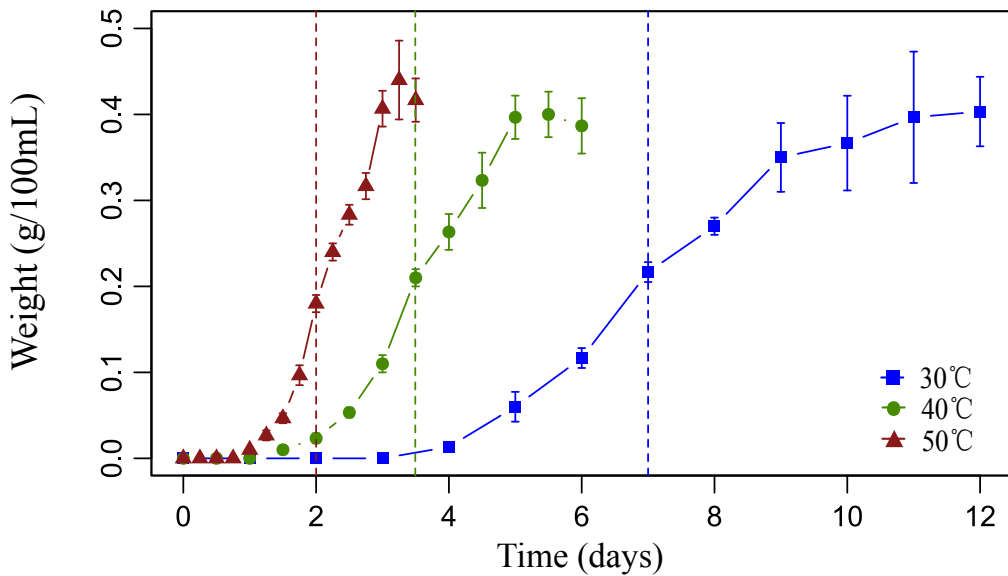

Growth on R<sub>2</sub>A-CMC media

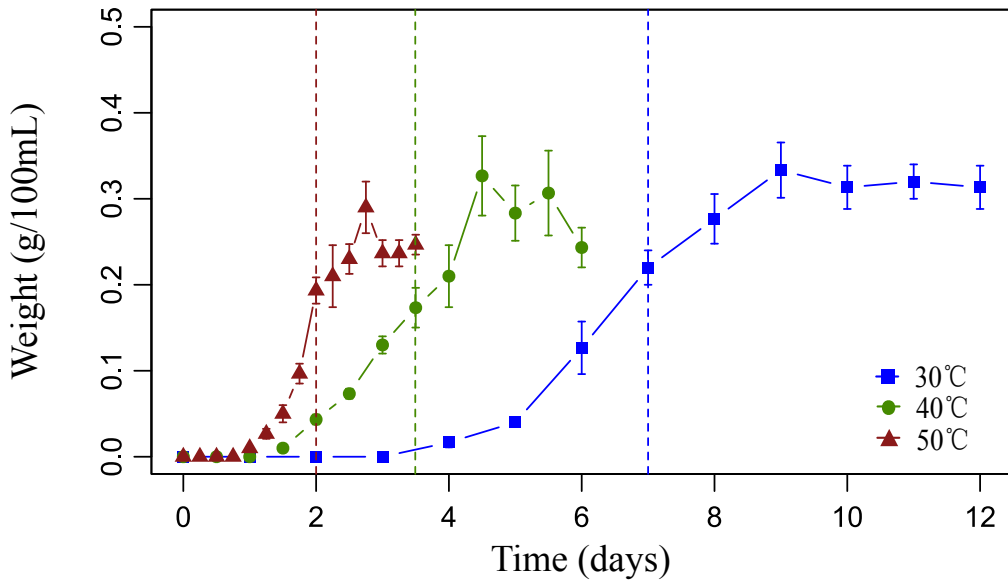

Supplement: Supplementary file 3 [file Presentation3.PDF]

# Pearson correlation between samples

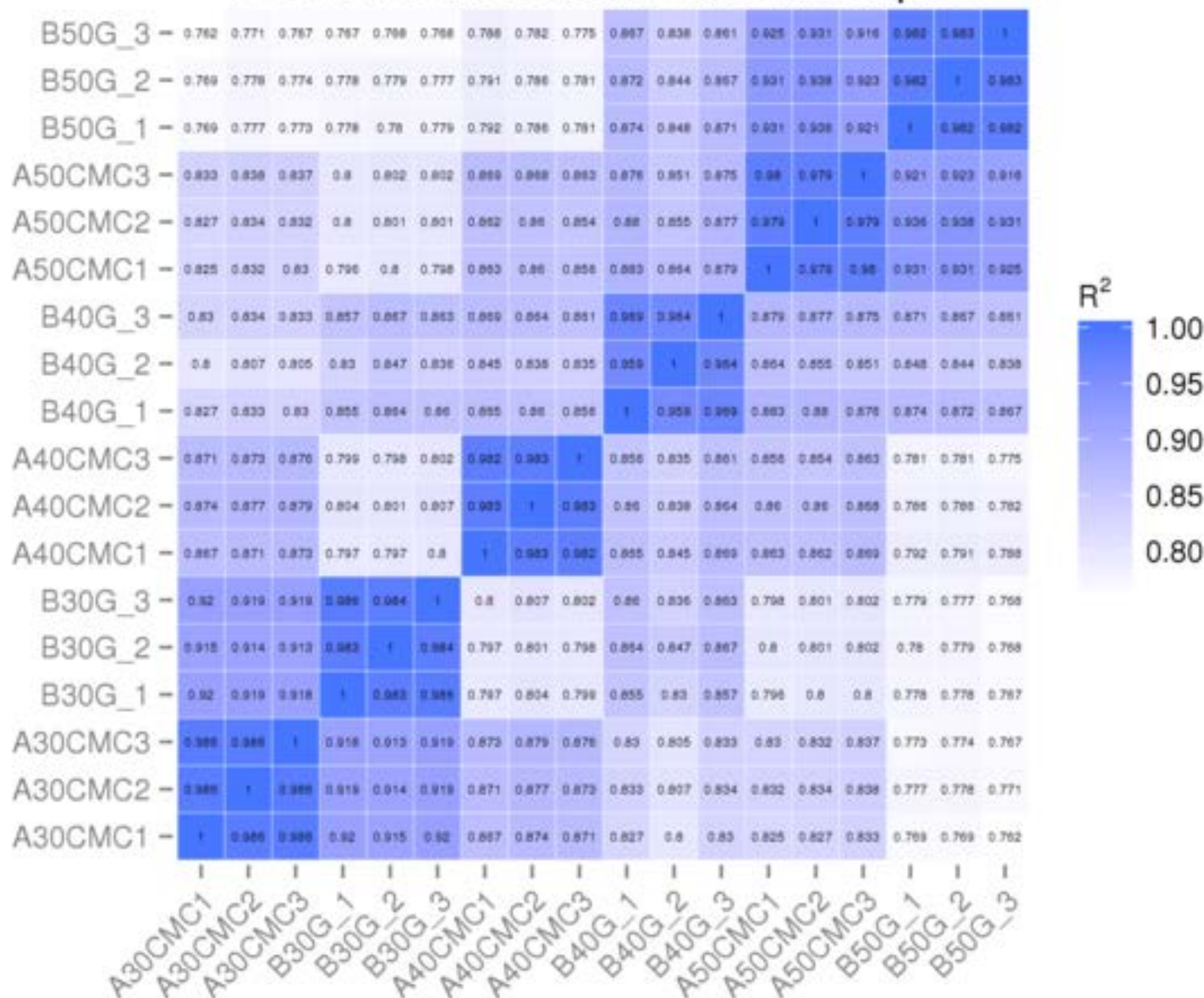

Supplement: Supplementary file 4 [file Presentation4.PDF]

Percent of Reads Mapped to Intergenic Regions (%)

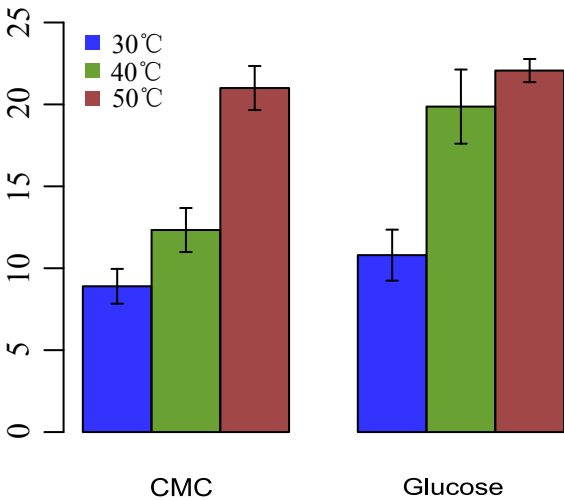

Supplement: Supplementary file 5 [file Presentation5.PDF]

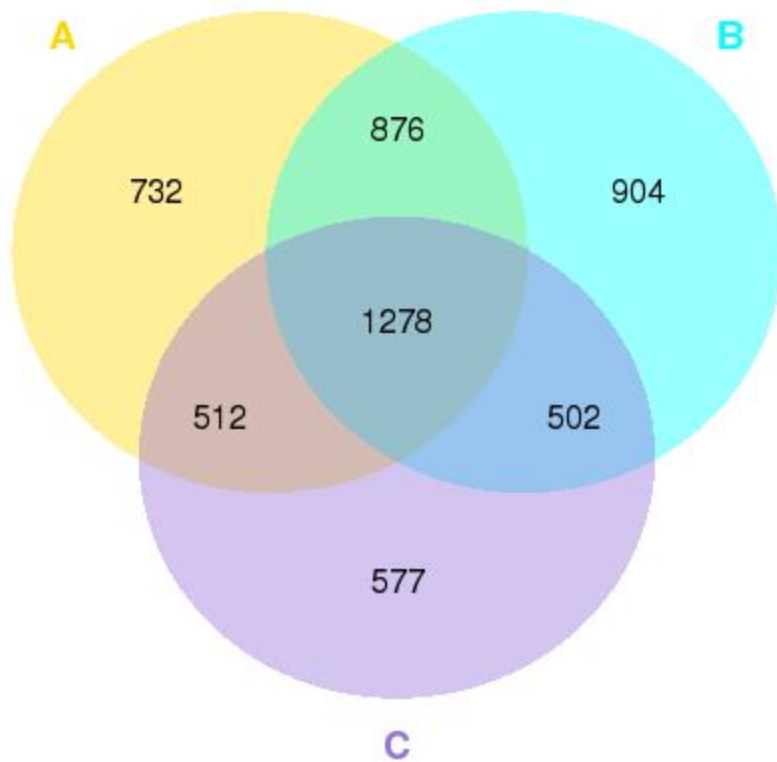

A: A30CMCvsB30G

B: A40CMCvsB40G

C: A50CMCvsB50G

Supplement: Supplementary file 7 [file Presentation7.PDF]

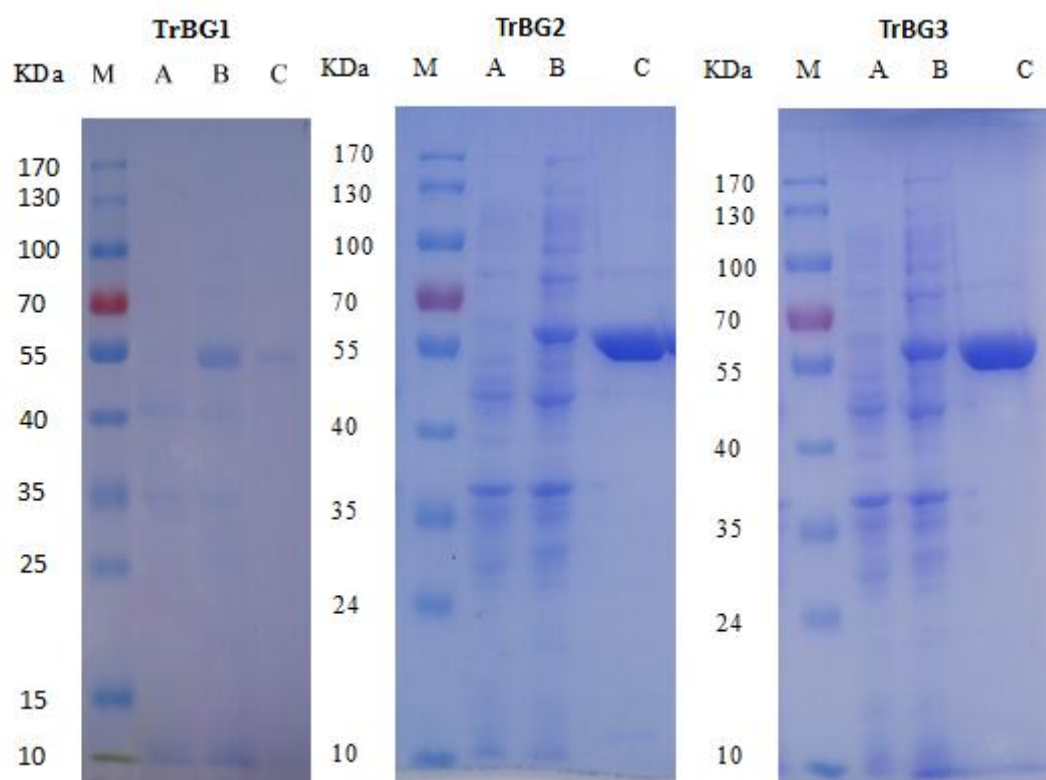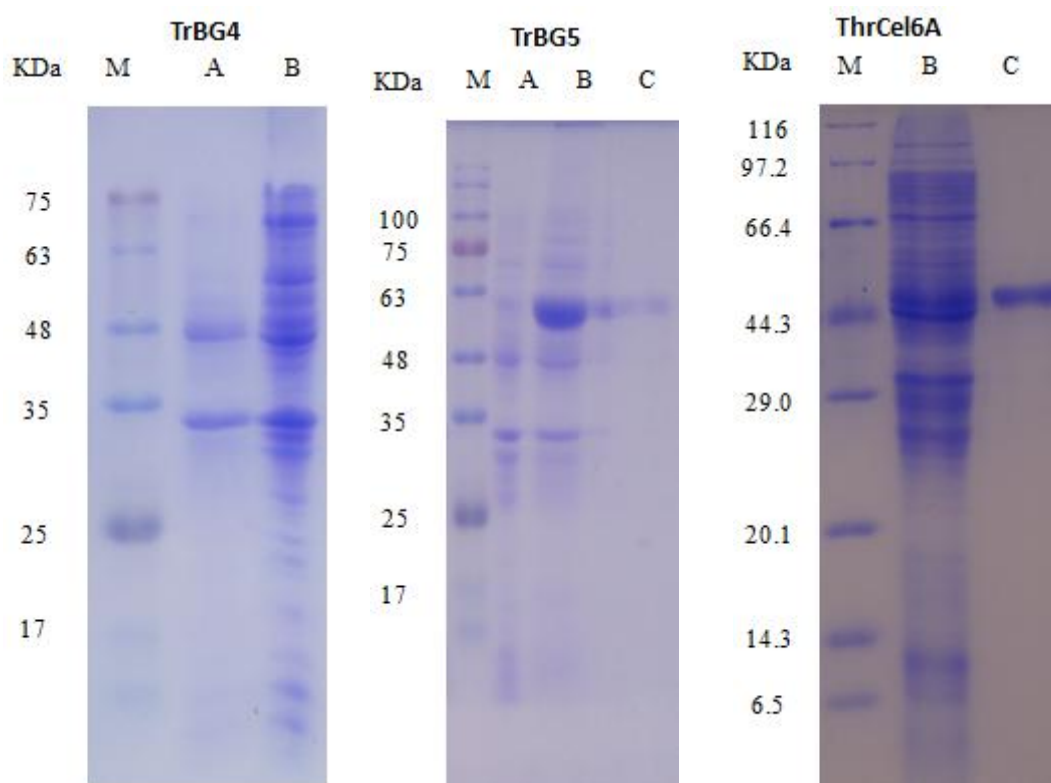

Supplement: Supplementary file 8 [file Presentation8.PDF]

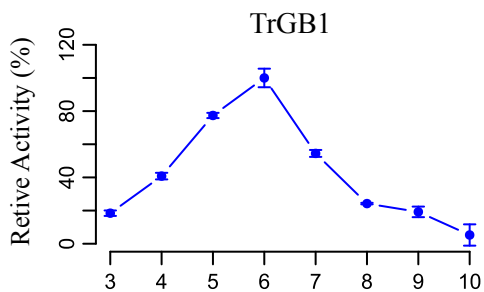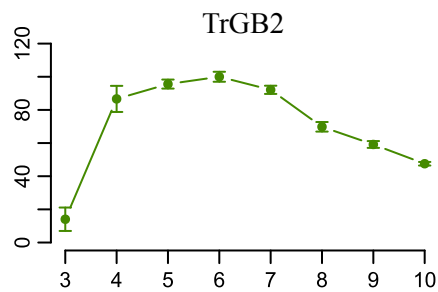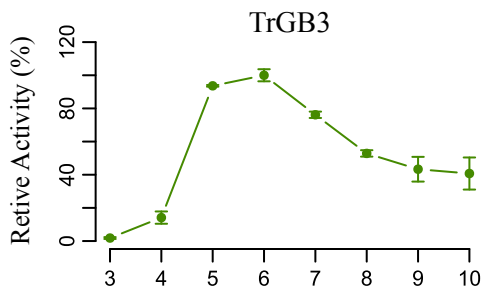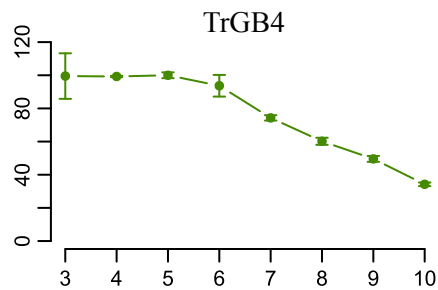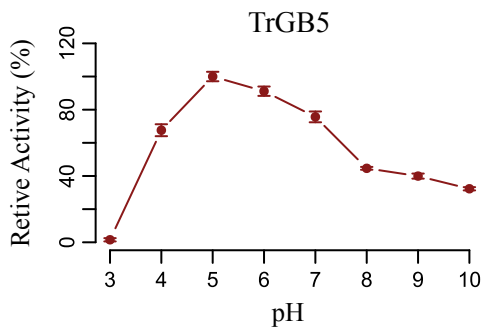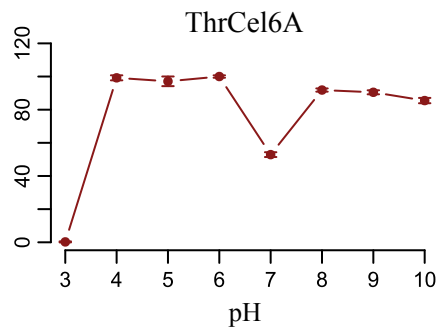

Supplement: Supplementary file 9 [file Presentation9.PDF]

A

## Cold shock protein (CSP)

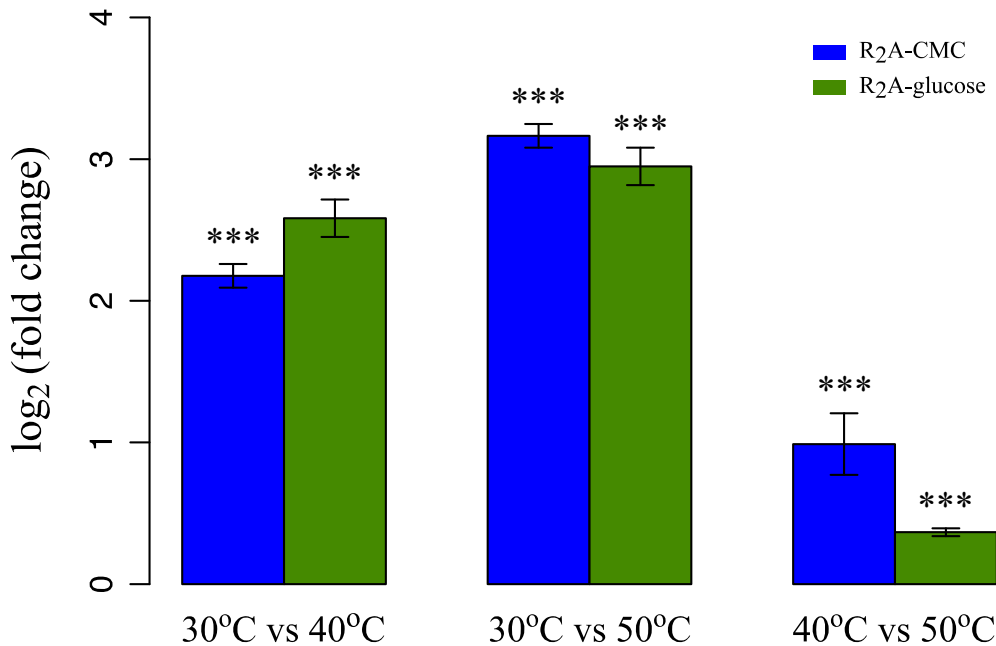

B

# Heat shock protein (HSP)

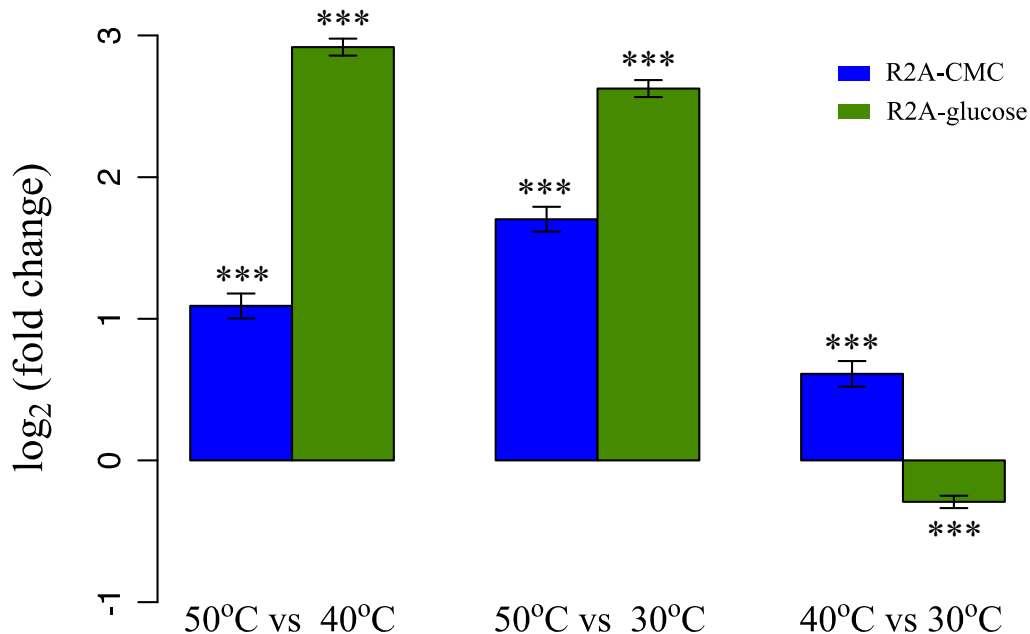

Supplement: Supplementary file 10 [file Presentation10.PDF]
