# Supplementary material for: The Hybrid Strategy of Thermoactinospora rubra YIM 77501T for Utilizing Cellulose as a Carbon Source at Different Temperatures
Source: Front Microbiol. 2017 May 29;8:942. doi: 10.3389/fmicb.2017.00942 (PMC5447088; doi:10.3389/fmicb.2017.00942)
Supplement: Supplementary file 6 [file Presentation6.PDF]

A

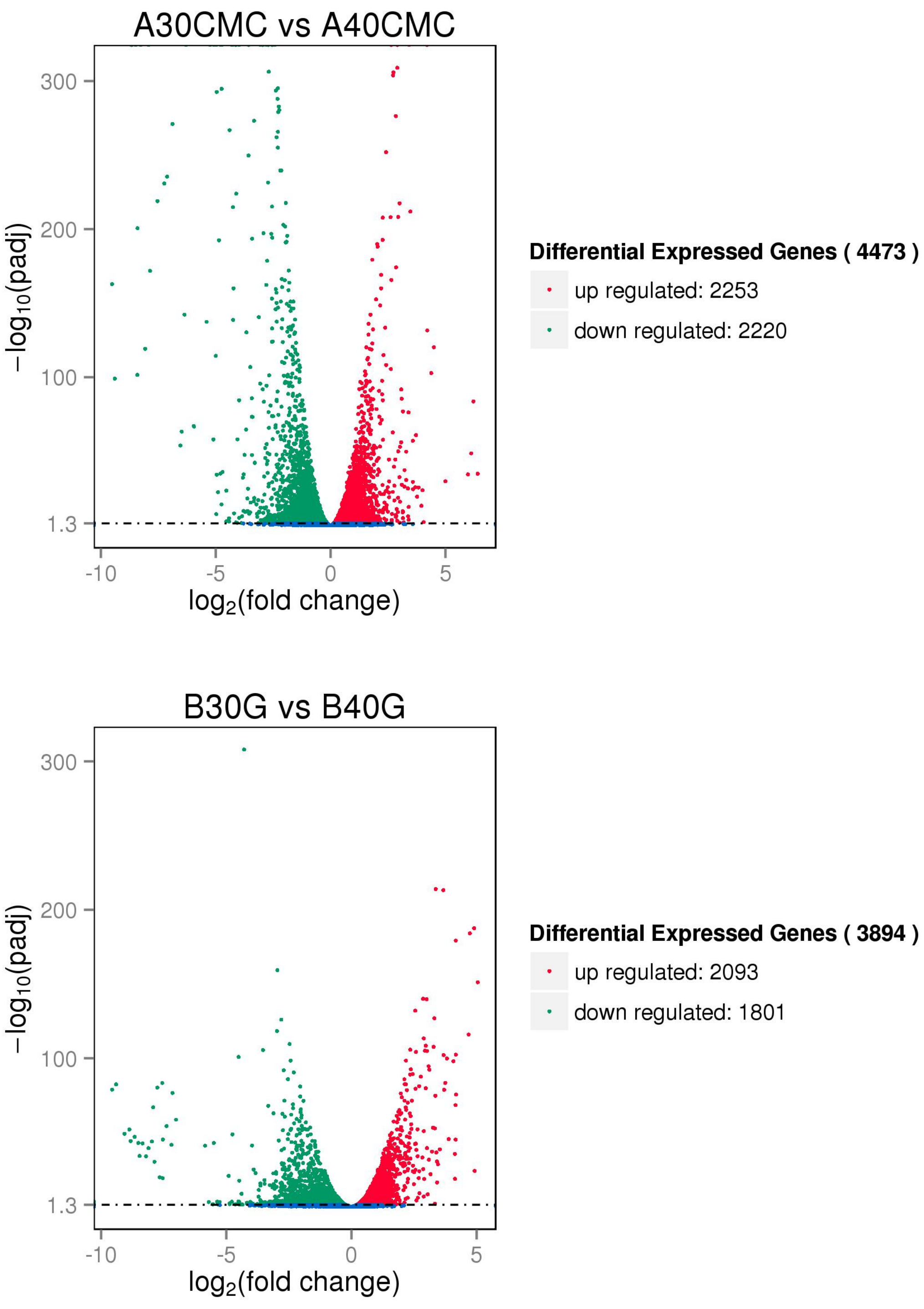
A30CMC vs A40CMC
300
200
100
1.3
-10
-5
0
5
−log10(padj)
log2(fold change)
Differential Expressed Genes (4473)
up regulated: 2253
down regulated: 2220
B30G vs B40G
300
200
100
1.3
-10
-5
0
5
−log10(padj)
log2(fold change)
Differential Expressed Genes (3894)
up regulated: 2093
down regulated: 1801

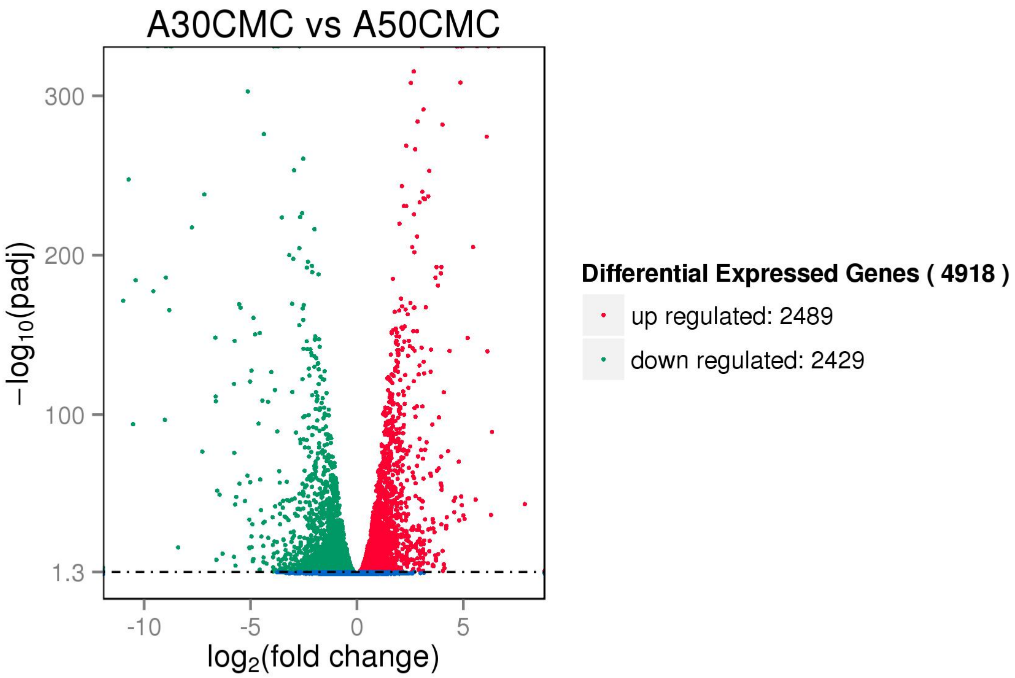
A30CMC vs A50CMC
300
200
100
1.3
−log10(padj)
-10
-5
0
5
log2(fold change)
Differential Expressed Genes (4918)
up regulated: 2489
down regulated: 2429

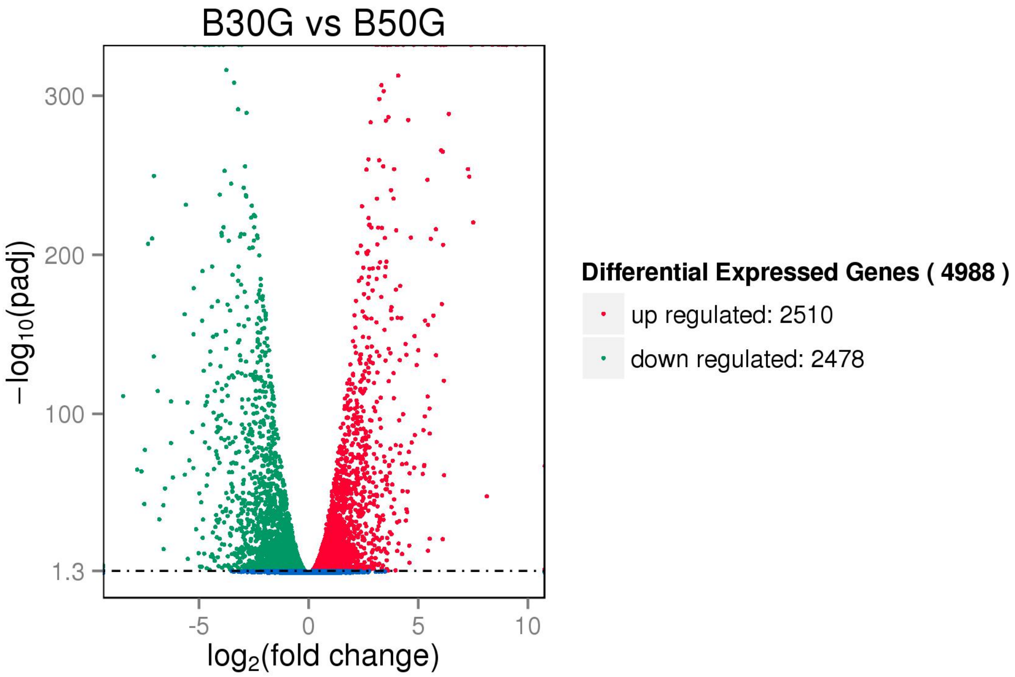
B30G vs B50G
300
200
100
1.3
−log10(padj)
-5
0
5
10
log2(fold change)
Differential Expressed Genes (4988)
up regulated: 2510
down regulated: 2478

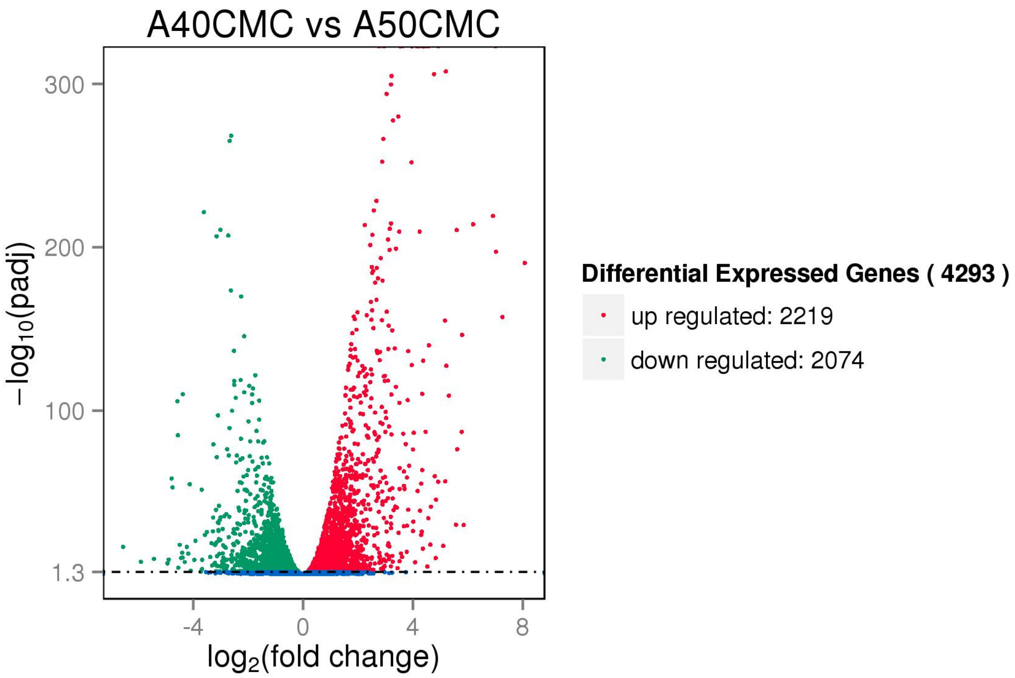
A40CMC vs A50CMC
300
200
100
1.3
−log10(padj)
-4
0
4
8
log2(fold change)
Differential Expressed Genes (4293)
up regulated: 2219
down regulated: 2074

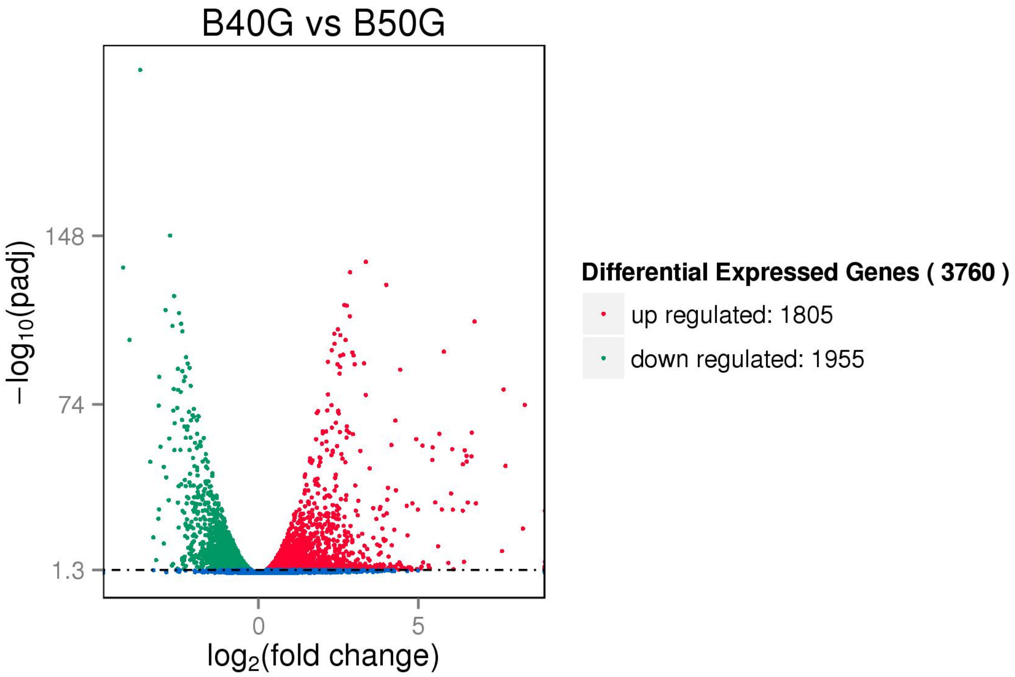
B40G vs B50G
148
74
1.3
−log10(padj)
0
5
log2(fold change)
Differential Expressed Genes (3760)
up regulated: 1805
down regulated: 1955

B

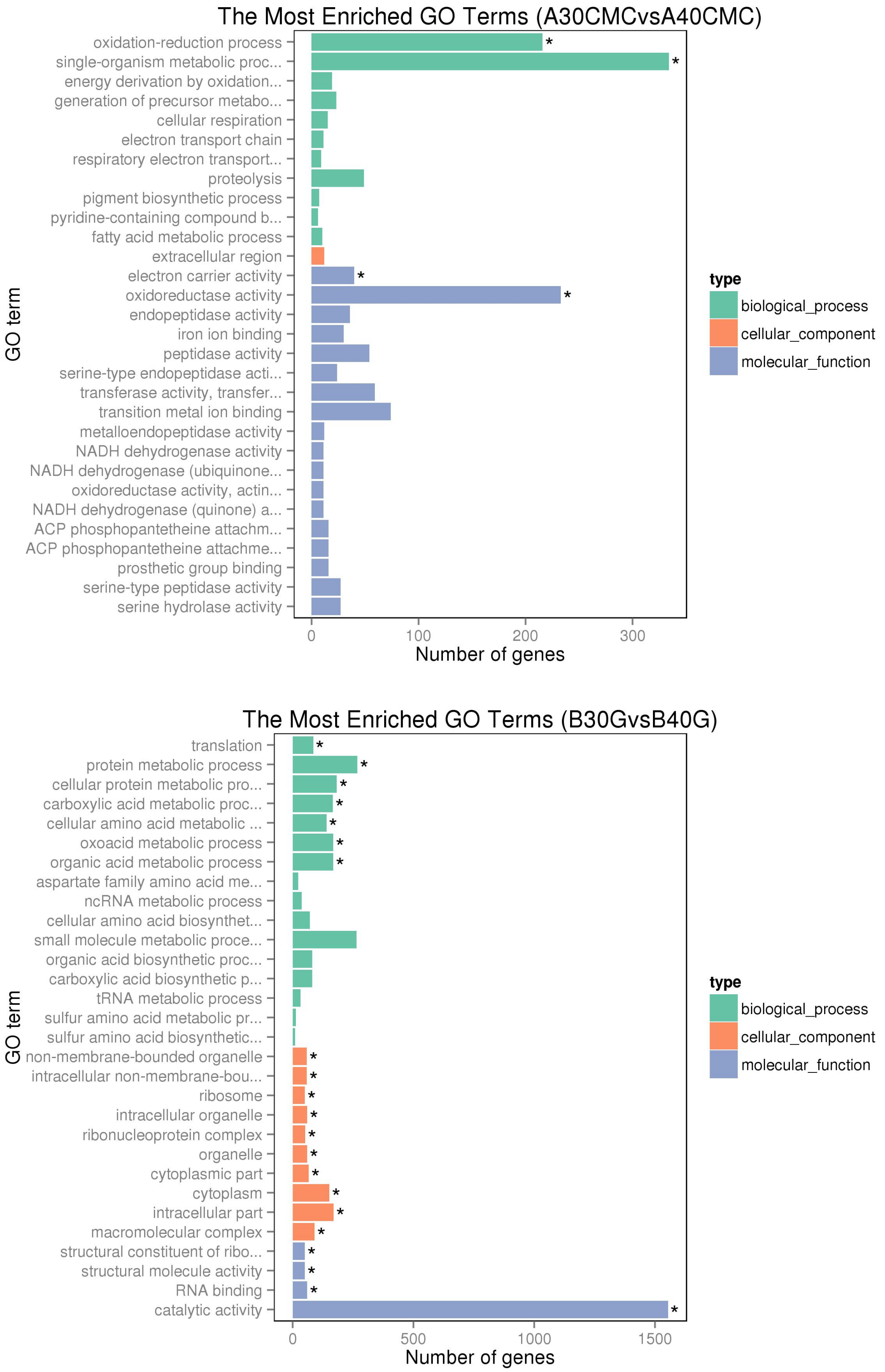
The Most Enriched GO Terms (A30CMCvsA40CMC)
oxidation-reduction process
single-organism metabolic proc...
energy derivation by oxidation...
generation of precursor metabo...
cellular respiration
electron transport chain
respiratory electron transport...
proteolysis
pigment biosynthetic process
pyridine-containing compound b...
fatty acid metabolic process
extracellular region
electron carrier activity
oxidoreductase activity
endopeptidase activity
iron ion binding
peptidase activity
serine-type endopeptidase acti...
transferase activity, transfer...
transition metal ion binding
metalloendopeptidase activity
NADH dehydrogenase activity
NADH dehydrogenase (ubiquinone...
oxidoreductase activity, actin...
NADH dehydrogenase (quinone) a...
ACP phosphopantetheine attachm...
ACP phosphopantetheine attachme...
prosthetic group binding
serine-type peptidase activity
serine hydrolase activity
GO term
0
100
200
300
Number of genes
type
biological_process
cellular_component
molecular_function
The Most Enriched GO Terms (B30GvsB40G)
translation
protein metabolic process
cellular protein metabolic pro...
carboxylic acid metabolic proc...
cellular amino acid metabolic ...
oxoacid metabolic process
organic acid metabolic process
aspartate family amino acid me...
ncRNA metabolic process
cellular amino acid biosynthet...
small molecule metabolic proce...
organic acid biosynthetic proc...
carboxylic acid biosynthetic p...
tRNA metabolic process
sulfur amino acid metabolic pr...
sulfur amino acid biosynthetic...
non-membrane-bounded organelle
intracellular non-membrane-bou...
ribosome
intracellular organelle
ribonucleoprotein complex
organelle
cytoplasmic part
cytoplasm
intracellular part
macromolecular complex
structural constituent of ribo...
structural molecule activity
RNA binding
catalytic activity
GO term
0
500
1000
1500
Number of genes
type
biological_process
cellular_component
molecular_function

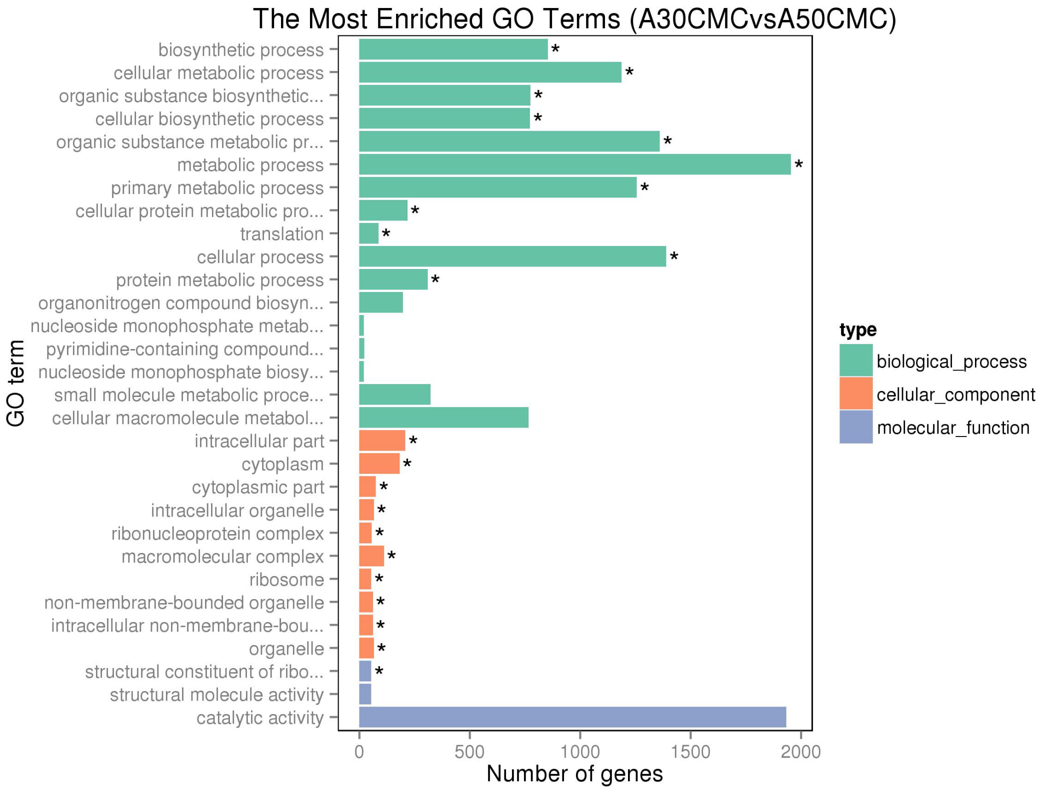

The Most Enriched GO Terms (A30CMCvsA50CMC)
biosynthetic process
cellular metabolic process
organic substance biosynthetic...
cellular biosynthetic process
organic substance metabolic pr...
metabolic process
primary metabolic process
cellular protein metabolic pro...
translation
cellular process
protein metabolic process
organonitrogen compound biosyn...
nucleoside monophosphate metab...
pyrimidine-containing compound...
nucleoside monophosphate biosy...
small molecule metabolic proce...
cellular macromolecule metabol...
intracellular part
cytoplasm
cytoplasmic part
intracellular organelle
ribonucleoprotein complex
macromolecular complex
ribosome
non-membrane-bounded organelle
intracellular non-membrane-bou...
organelle
structural constituent of ribo...
structural molecule activity
catalytic activity
GO term
0
500
1000
1500
2000
Number of genes
type
biological_process
cellular_component
molecular_function

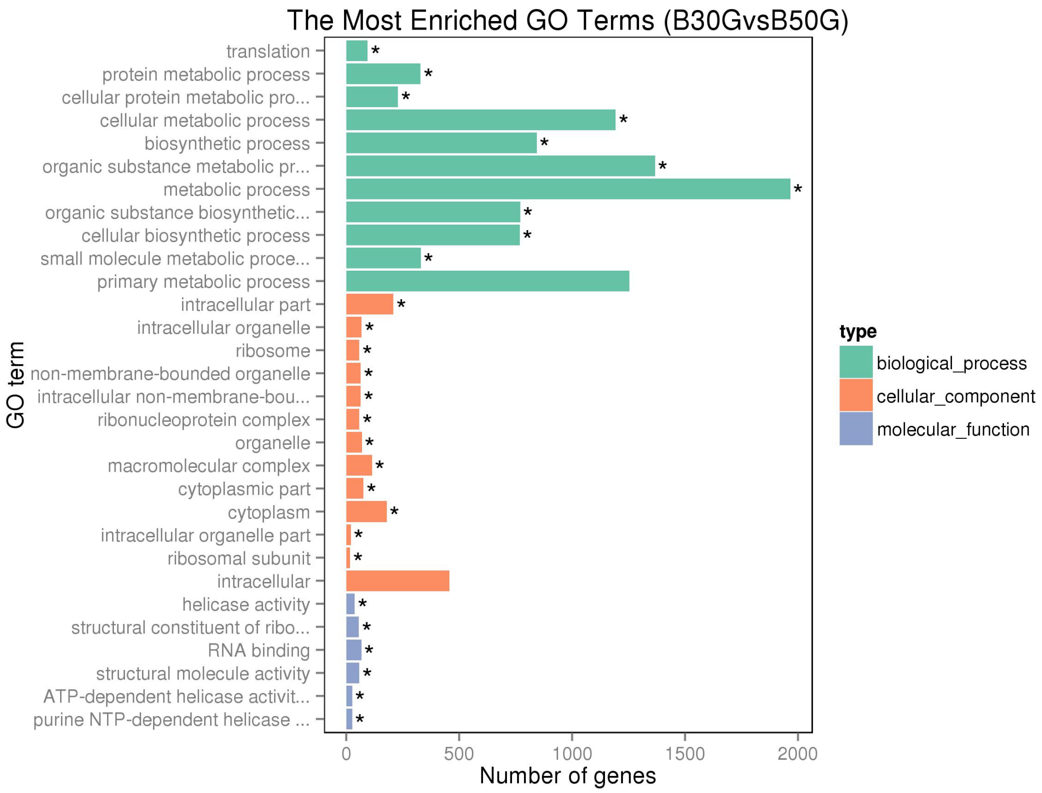

The Most Enriched GO Terms (B30GvsB50G)
translation
protein metabolic process
cellular protein metabolic pro...
cellular metabolic process
biosynthetic process
organic substance metabolic pr...
metabolic process
organic substance biosynthetic...
cellular biosynthetic process
small molecule metabolic proce...
primary metabolic process
intracellular part
intracellular organelle
ribosome
non-membrane-bounded organelle
intracellular non-membrane-bou...
ribonucleoprotein complex
organelle
macromolecular complex
cytoplasmic part
cytoplasm
intracellular organelle part
ribosomal subunit
intracellular
helicase activity
structural constituent of ribo...
RNA binding
structural molecule activity
ATP-dependent helicase activit...
purine NTP-dependent helicase ...
GO term
0
500
1000
1500
2000
Number of genes
type
biological_process
cellular_component
molecular_function

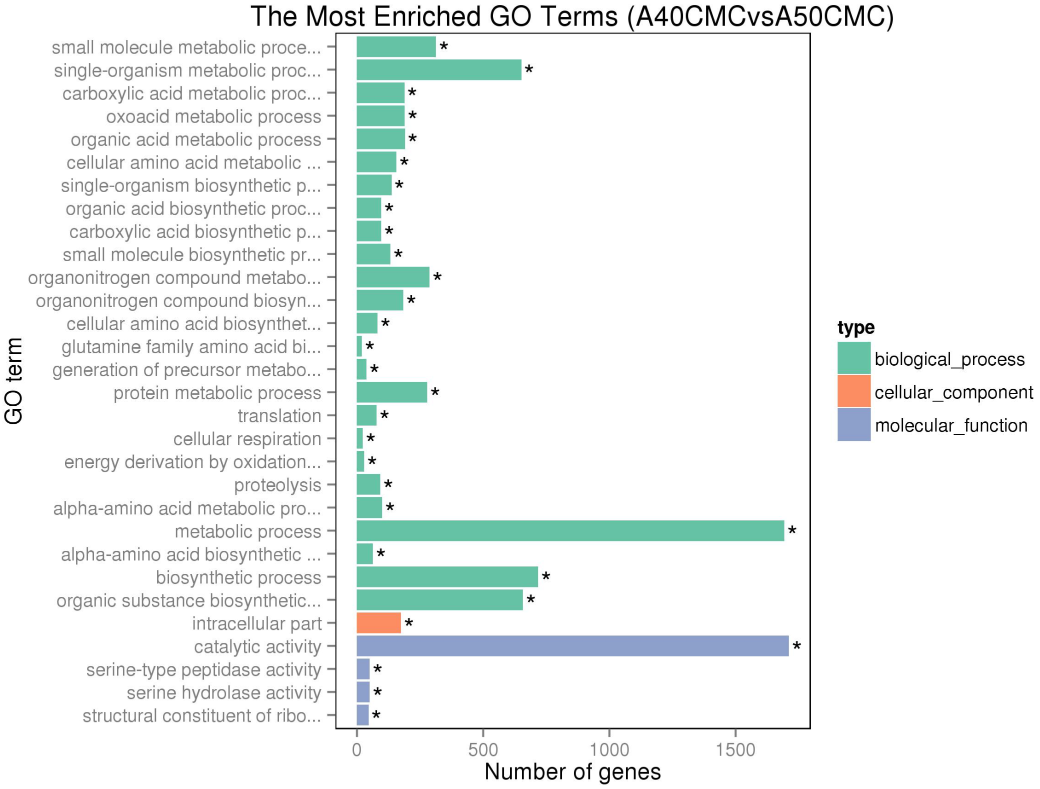
The Most Enriched GO Terms (A40CMCvsA50CMC)
small molecule metabolic proce...
single-organism metabolic proc...
carboxylic acid metabolic proc...
oxoacid metabolic process
organic acid metabolic process
cellular amino acid metabolic ...
single-organism biosynthetic p...
organic acid biosynthetic proc...
carboxylic acid biosynthetic p...
small molecule biosynthetic pr...
organonitrogen compound metabo...
organonitrogen compound biosyn...
cellular amino acid biosynthet...
glutamine family amino acid bi...
generation of precursor metabo...
protein metabolic process
translation
cellular respiration
energy derivation by oxidation...
proteolysis
alpha-amino acid metabolic pro...
metabolic process
alpha-amino acid biosynthetic ...
biosynthetic process
organic substance biosynthetic...
intracellular part
catalytic activity
serine-type peptidase activity
serine hydrolase activity
structural constituent of ribo...
GO term
0
500
1000
1500
Number of genes
type
biological_process
cellular_component
molecular_function

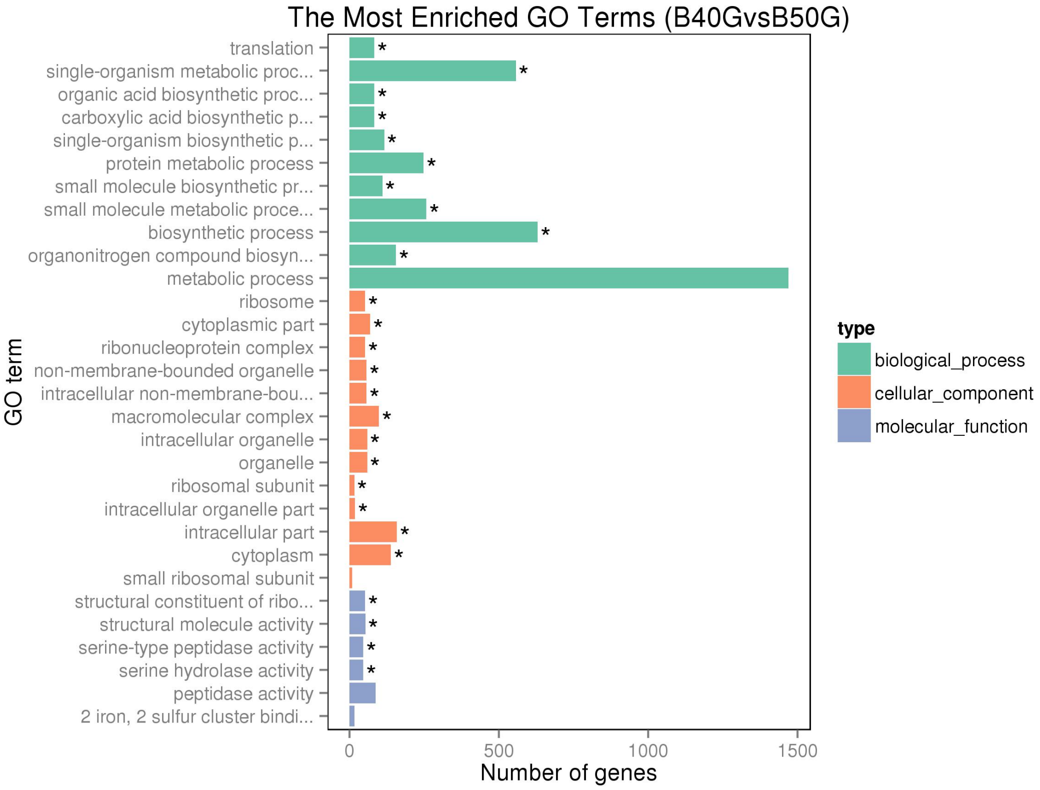
The Most Enriched GO Terms (B40GvsB50G)
translation
single-organism metabolic proc...
organic acid biosynthetic proc...
carboxylic acid biosynthetic p...
single-organism biosynthetic p...
protein metabolic process
small molecule biosynthetic pr...
small molecule metabolic proce...
biosynthetic process
organonitrogen compound biosyn...
metabolic process
ribosome
cytoplasmic part
ribonucleoprotein complex
non-membrane-bounded organelle
intracellular non-membrane-bou...
macromolecular complex
intracellular organelle
organelle
ribosomal subunit
intracellular organelle part
intracellular part
cytoplasm
small ribosomal subunit
structural constituent of ribo...
structural molecule activity
serine-type peptidase activity
serine hydrolase activity
peptidase activity
2 iron, 2 sulfur cluster bindi...
GO term
0
500
1000
1500
Number of genes
type
biological_process
cellular_component
molecular_function
