# Supplementary material for: The Hybrid Strategy of Thermoactinospora rubra YIM 77501T for Utilizing Cellulose as a Carbon Source at Different Temperatures
Source: Front Microbiol. 2017 May 29;8:942. doi: 10.3389/fmicb.2017.00942 (PMC5447088; doi:10.3389/fmicb.2017.00942)
Supplement: Supplementary file 11 [file Table1.DOCX]

Supplementary material of “**The hybrid strategy of *Thermoactinospora rubra* for utilizing cellulose as a carbon source at different temperatures**”

List of Supplementary Information

Five TABLEs:

**TABLE S1** Primers used in this study.

**TABLE S2** General features of the genome of *T. rubra* YIM 77501^T^.

**TABLE S3** Summary of data generated in the transcriptome sequence of *T.* YIM 77501^T^.

**TABLE S4** Number of different gene expression level (FPKM interval)

**TABLE S5** The kinetic parameters of up-regulated cellulases

Ten Figures:

**FIG S1** Plate testing for cellulase production of strain *T. rubra* YIM 77501^T^ using Congo red plate assay. After strain *T. rubra* YIM 77501^T^ culturing in R_2_A-CMC agar media for 48 h at 45 °C, the clear haloes appeared around colony.

**FIG S2** The cellulases activity of culture supernatants of strain *T. rubra* YIM 77501^T^ which cultivated in 0.2% R_2_A-CMC media to stationary phase at 30 °C, 40 °C and 50 °C, respectively. All assays were performed in triplicate, and standard deviations are shown with *error bars*.

**FIG S3** The growth curve of strain *Thermoactinospora rubra* YIM 77501^T^ cultured. **(A)** Strain *T. rubra* YIM 77501^T^ cultured in R2A-glucose media at 30 °C, 40 °C and 50 °C, respectively.**(B)** Strain *T. rubra* YIM 77501^T^ cultured in R2A-CMC media at 30 °C, 40 °C and 50 °C, respectively. Dotted lines show the time of mid exponential phase at different cultural conditions. All assays were performed in triplicate, and standard deviations are shown with *error bars*.

**FIG S4** Pearson correlation between samples.

**FIG S5** Percent of reads mapped to intergenic regions. “CMC” means strain *T. rubra* YIM 77501^T^ was cultured in CMC-R2A media. “Glucose” means strain was cultured in Glucose-R2A media. Colors of histogram mean strain *T. rubra* YIM 77501^T^ was incubated at temperatures 30 °C (blue), 40°C (green), 50°C (red), respectively.

**FIG S6** Comparing genes expression at different temperature. **(A)** Volcanic figure of differential expressed genes at different temperature. Red points show up-regulated genes. Green points show down-regulated genes. **(B)** The most enriched GO terms. "*" indicates significant enrichment of GO term. A30CMC, A40CMC and A50CMC mean that data collected from strain *T. rubra* YIM 77501^T^ which cultured at CMC media at 30 °C, 40 °C and 50 °C, respectively. B30G, B40G and B50G mean that data collected from strain *T. rubra* YIM 77501^T^ which cultured at glucose media at 30 °C, 40 °C and 50 °C, respectively.

**FIG S7** Venn diagram of up- and down- regulated genes. The Venn diagram shows the numbers of the change of gene expression in present of CMC (A30CMC, A40CMC and A50CMC), culture in glucose media as control (B30G, B40G and B50G). The color of orange, green and blue mean culture at 30 °C, 40 °C and 50 °C, respectively.

**FIG S8** Analysis of the recombinant proteins: TrBG1, TrBG2, TrBG3, TrBG4, TrBG5and ThrCel6A on SDS-PAGE. Lane M, protein molecular weight marker (kDa); lane A, total protein in non-induced *E.coli* BL21/*pEASY*-Blunt E1-*ThrGH1A*; lane B, total protein in IPTG-induced *E.coli* BL21/*pEASY*-Blunt E1-*ThrGH1A*; lane C, purified ThrGH1A.

**FIG S9** The optimal reaction pH of TrBG1, TrBG2, TrBG3, ThrCel6B, TrBG4, TrBG5and ThrCel6A. The effect of pH on enzymes were investigated in buffer ranging from pH 3.0 to 10 by using the following buffer: sodium citrate (pH 3.0–6.0), Tris–HCl (pH 7.0–8.0); glycine-NaOH (pH9.0–10.5). For TrBG1, 100%=0.075 U/mg; TrBG2, 100%= 33.4 U/mg; TrBG3, 100%=0.86 U/mg; TrBG4, no data; TrBG5, 100%=6.6 U/mg; ThrCel6A, 100%=5.3 U/mg. All assays were performed in triplicate, and standard deviations are shown with *error bars*.

**FIG S10** Log2 of fold change in gene expression level of cold shock protein (CSP) and heat shock protein (HSP) for Strain *T. rubra* YIM 77501^T^ cultured at different temperatures (30 °C, 40 °C and 50 °C). (A) Cold shock protein (CSP), (B) Heat shock protein (HSP), also named DnaK. R_2_A-CMCand R_2_A-Glucose are cultured media of *T. rubra* YIM 77501^T^. *** Significant at *p* < 0.01; ** Significant at *p*< 0.05; * Significant at *p*< 0.1.

**TABLES1. Primers used in this study.**

| **Name** | **Sequence (5′—3′)** |
| --- | --- |
| 0715-F | GTGACCTCGCAGGAG |
| 0715-R | TTATTCTGCCGCAACG |
| 0649-F | ATGAGTTTTCTGTGGG |
| 0649-R | TCAGCCGAGCCCACCT |
| 7-003570-F | GTGCTCGCCCTCTTAGCCTCCG |
| 7-003570-R | CTACACGGCCTGGGCCAGCAG |
| 7-004020-F | GCCTGCGTGGCGGCCCTCGGTCT |
| 7—004020-R | TCAGTTCAACGGCGGGTAGGCGTTGCG |
| 7-002722-F | ATGCACACGTTTATCTGGGGT |
| 7-002722-R | TCATGTGCTGATGCGTTCCCTG |
| 0221--F | GTGGCGGCGCTCTCGCT |
| 0221-R | TCACCTGGCGACGCAGGTG |
| 006941-F1 | GTCGCCGGATTCCTCCTCCT |
| 006941-R1 | TGGGCTTCGTCTCCTGGGTC |
| 7-006941-F2 | GTGTCGCACTCCATCGACCTCGCCG |
| 7-006941-R2 | TCACGCGCCGCGGTGCTCCCG |

**TABLE S2. General features of the genome of *T. rubra* YIM 77501^T^.**

| **Parameter** | **value** |
| --- | --- |
| Size (bp) | 8,233,369 |
| Scaffold | 191 |
| G+C content (%) | 71.71% |
| protein coding genes |  |
| No. similar to known proteins (%) | 4867 (59.98) |
| No. similar to proteins of unknown function (%) | 1219 (15.02) |
| No. of hypothetical protein (%) | 2028 (24.99) |
| Total | 8,114 |
| Average ORF size (bp) | 914 |
| Coding (%) | 90.1 |
| No. of rRNA clusters | 1 |
| No. of tRNA genes | 54 |
| CAZy (Carbohydrate-Active EnZymes)genes | 1,237（rules）or 1,303 (orthology) |
| CAZy % of coding genes | 15.25% or 16.06% |

**TABLE S3.** **Summary of data generated in the transcriptome sequence of *T.* YIM 77501^T^.**

| **Sample name** | **Raw reads** | **Cleanreads** | **Cleanbases** | **Error(%)** | **Q20(%)** | **Q30(%)** | **GC(%)** |
| --- | --- | --- | --- | --- | --- | --- | --- |
| A30CMC_1_ | 22384038 | 21408030 | 2.68G | 0.01 | 98.48 | 95.65 | 68.86 |
| A30CMC_2_ | 21230216 | 20314018 | 2.54G | 0.01 | 98.49 | 95.66 | 68.75 |
| A30CMC_3_ | 21921860 | 20941388 | 2.62G | 0.01 | 98.37 | 95.38 | 68.71 |
| B30G_1_ | 22607780 | 21594402 | 2.7G | 0.01 | 98.48 | 95.64 | 69.00 |
| B30G_2_ | 21955996 | 20916936 | 2.61G | 0.01 | 98.54 | 95.77 | 69.18 |
| B30G_3_ | 23906872 | 22815220 | 2.85G | 0.01 | 98.58 | 95.87 | 69.05 |
| A40CMC_1_ | 26120348 | 24879264 | 3.11G | 0.01 | 98.52 | 95.73 | 68.43 |
| A40CMC_2_ | 25838920 | 24611078 | 3.08G | 0.01 | 98.34 | 95.30 | 68.45 |
| A40CMC_3_ | 23762382 | 22652752 | 2.83G | 0.01 | 98.55 | 95.79 | 68.50 |
| B40G_1_ | 23294454 | 22000124 | 2.75G | 0.01 | 98.64 | 96.03 | 68.56 |
| B40G_2_ | 25677120 | 24122714 | 3.02G | 0.01 | 98.61 | 95.94 | 68.37 |
| B40G_3_ | 24160024 | 22907882 | 2.86G | 0.01 | 98.55 | 95.80 | 68.57 |
| A50CMC_1_ | 24762860 | 23555666 | 2.94G | 0.01 | 98.58 | 95.87 | 68.57 |
| A50CMC_2_ | 23869714 | 22741106 | 2.84G | 0.01 | 98.58 | 95.88 | 68.49 |
| A50CMC_3_ | 24166600 | 23039294 | 2.88G | 0.01 | 98.51 | 95.71 | 68.23 |
| B50G_1_ | 22716898 | 21589942 | 2.7G | 0.01 | 98.56 | 95.82 | 68.79 |
| B50G_2_ | 23327088 | 22085298 | 2.76G | 0.01 | 98.61 | 95.95 | 68.61 |
| B50G_3_ | 25524654 | 24305586 | 3.04G | 0.01 | 98.68 | 96.11 | 68.68 |

A30CMC, A40CMC and A50CMC mean that data collected from strain ***T.rubra*YIM 77501^T^** which cultured at CMC media at **30 °C, 40 °C and 50 °C,** respectively. B30G, B40G and B50G mean that data collected from strain ***T.rubra*YIM 77501^T^** which cultured at glucose media at **30 °C, 40 °C and 50 °C,** respectively. The subscript Arabic numerals (_1, 2_ and _3_) mean three independent biological replicates.

**TABLE S4. Number of different gene expression level (FPKM interval).**

| **FPKM Interval** | **0~1** | **1~3** | **3~15** | **15~60** | **>60** |
| --- | --- | --- | --- | --- | --- |
| A30CMC_1_ | 1034(12.74%) | 753(9.28%) | 1577(19.44%) | 2019(24.88%) | 2731(33.66%) |
| A30CMC_2_ | 1010(12.45%) | 749(9.23%) | 1590(19.60%) | 2026(24.97%) | 2739(33.76%) |
| A30CMC_3_ | 1046(12.89%) | 739(9.11%) | 1585(19.53%) | 2037(25.10%) | 2707(33.36%) |
| B30G_1_ | 1081(13.32%) | 802(9.88%) | 1548(19.08%) | 1950(24.03%) | 2733(33.68%) |
| B30G_2_ | 796(9.81%) | 901(11.10%) | 1678(20.68%) | 1953(24.07%) | 2786(34.34%) |
| B30G_3_ | 1069(13.17%) | 808(9.96%) | 1578(19.45%) | 1928(23.76%) | 2731(33.66%) |
| A40CMC_1_ | 1047(12.90%) | 847(10.44%) | 1826(22.50%) | 2171(26.76%) | 2223(27.40%) |
| A40CMC_2_ | 1146(14.12%) | 800(9.86%) | 1802(22.21%) | 2127(26.21%) | 2239(27.59%) |
| A40CMC_3_ | 1122(13.83%) | 833(10.27%) | 1816(22.38%) | 2112(26.03%) | 2231(27.50%) |
| B40G_1_ | 424(5.23%) | 830(10.23%) | 2127(26.21%) | 2141(26.39%) | 2592(31.94%) |
| B40G_2_ | 311(3.83%) | 387(4.77%) | 2418(29.80%) | 2442(30.10%) | 2556(31.50%) |
| B40G_3_ | 608(7.49%) | 924(11.39%) | 1994(24.57%) | 2106(25.96%) | 2482(30.59%) |
| A50CMC_1_ | 575(7.09%) | 787(9.70%) | 1763(21.73%) | 2146(26.45%) | 2843(35.04%) |
| A50CMC_2_ | 865(10.66%) | 686(8.45%) | 1609(19.83%) | 2107(25.97%) | 2847(35.09%) |
| A50CMC_3_ | 867(10.69%) | 705(8.69%) | 1585(19.53%) | 2140(26.37%) | 2817(34.72%) |
| B50G_1_ | 904(11.14%) | 761(9.38%) | 1622(19.99%) | 1964(24.21%) | 2863(35.28%) |
| B50G_2_ | 883(10.88%) | 774(9.54%) | 1625(20.03%) | 1961(24.17%) | 2871(35.38%) |
| B50G_3_ | 870(10.72%) | 804(9.91%) | 1627(20.05%) | 1991(24.54%) | 2822(34.78%) |

FPKM, expected number of Fragments Per Kilobase of transcript sequence per Millions base pairs sequenced, considers the effect of sequencing depth and gene length for the reads count at the same time, and is currently the most commonly used method for estimating gene expression levels. Here, FPKM > 1 means gene expression.

**TABLE S5. The kinetic parameters of up-regulated cellulases.**

|  | **TrBG2** | **TrBG3** | **TrBG4** | **ThrCel6B** | | **TrBG1** | **TrBG5** | **ThrCel6A** |
| --- | --- | --- | --- | --- | --- | --- | --- | --- |
| Substrate | pNPGlu | pNPGlu | pNPGlu | | — | pNPGlu | pNPGlu | CMC |
| Optimal temperature (°C) | 60 | 50 | 60 | | — | 40 | 50 | 70 |
| Optimal pH | 6 | 6 | 3～5 | | — | 6 | 5 | 4～6 |
| Specific activity (U/mg) | 33.4±1.5 | 0.86±0.06 | — | | — | 0.075±0.002 | 6.6±0.4 | 5.3±0.5 |
| Km | 0.31 µmol/ml | 5.9 µmol/ml | — | | — | 9.8 µmol/ml | 1.4 µmol/ml | 47.7 mg/ml |
| Vmax (µmol/min/mg) | 43.5 | 2 | — | | — | 0.12 | 11 | 29.6 |
| Molecular mass (Da) | 50871.05 | 54206.64 | 107605 | | 82018 | 50940.15 | 51246.56 | 47225.17 |
| Kcat (s-1) | 36.9 | 1.8 | — | | — | 0.1 | 9.4 | 23.3 |
| signal peptide | NO | NO | YES | | YES | NO | NO | NO |

“—”means no data. “NO”means no signal peptide was discriminated by SignalP 4.1 Server (<http://www.cbs.dtu.dk/services/SignalP/>).

“YES” means signal peptide was discriminated by SignalP 4.1 Server.
